# Immune Evasion Strategies of Human Poxviruses: A Roadmap for Engineering Low-Immunogenic CAR-Based and iPSC-Derived Cell Products

**DOI:** 10.3390/cells15201902

**Published:** 2026-10-19

**Authors:** Daniil O. Antonov, Marina Yu. Loguinova, Hui Fan, Elena V. Sazonova, Anastasiia O. Sosnovtseva, Dmitry S. Karpov, Artem D. Molchanov, Vladimir N. Nikolenko, Vladimir P. Chekhonin, Natalia G. Mokrysheva

**Affiliations:** 1 Endocrinology Research Centre, 117036 Moscow, Russia; loginova.marina@endocrincentr.ru (M.Y.L.); fan.hui@endocrincentr.ru (H.F.); sazonova.elena@endocrincentr.ru (E.V.S.); molchanov.artem@endocrincentr.ru (A.D.M.); chehonin.vladimir@endocrincentr.ru (V.P.C.); mokrisheva.natalia@endocrincentr.ru (N.G.M.); 2 Department of Human Anatomy and Histology, N. V. Sklifosovsky Institute of Clinical Medicine, First Moscow State Medical University ( Sechenov University), 125009 Moscow, Russia; nikolenko_v_n@staff.sechenov.ru; 3 Vavilov Institute of General Genetics, Russian Academy of Sciences, 119991 Moscow, Russia; sosnovtceva@vigg.ru; 4 Engelhardt Institute of Molecular Biology, Russian Academy of Sciences, 119991 Moscow, Russia

**Keywords:** poxvirus immune evasion, allogeneic cell therapy, CAR-T cells, CAR-NK cells, iPSC-derived cell products, hypoimmune engineering, interferon signaling, antigen presentation, NK-cell surveillance, complement regulation

## Abstract

Allogeneic chimeric antigen receptor (CAR)-based cell products and induced pluripotent stem cell (iPSC)-derived regenerative grafts increasingly use multilayer immune engineering. Current strategies are strongest against direct human leukocyte antigen (HLA)-dependent recognition but incompletely address natural killer (NK)-cell counter-pressure, priming by recipient antigen-presenting cells, humoral and complement-mediated injury, inflammatory remodeling, blood- and tissue-interface damage, and terminal cell killing. Poxvirus research has characterized diverse immunomodulatory mechanisms relevant to these barriers. We review poxviral control of interferon signaling, antigen processing and presentation, T-cell activation, NK-cell surveillance, cytokine and chemokine networks, complement, inflammatory signaling, and regulated cell death, integrating viral mechanisms with human perturbation, cell-engineering, and transplantation evidence. A qualitative Tier 1–3 framework distinguishes mechanisms close to current cell-therapy practice from those requiring greater product-specific validation or control. The resulting design rules favor graded antigen-presentation control with paired NK validation, local or transient inflammatory buffering, interface-restricted complement protection, and survival enhancement only when independent eliminability is preserved. Selected poxviral proteins can act autonomously outside productive infection, but direct viral-protein expression remains a selective rather than default option. We propose a conditional defense-in-depth framework in which modules are added only when a residual barrier is demonstrated in the final therapeutic product.

## 1. Introduction

Allogeneic human induced pluripotent stem cell (iPSC)-derived grafts and allogeneic chimeric antigen receptor (CAR)-based therapies offer scalable approaches to tissue replacement and “off-the-shelf” cellular treatment [1,2,3,4]. Their broader use, however, is limited by recipient immune recognition, inflammatory injury, and loss of therapeutic-cell persistence or function [5,6,7]. These problems are not driven by one pathway. They arise from overlapping innate, adaptive, humoral, and tissue-stress responses.

The choice between autologous and allogeneic products involves a trade-off between immunological compatibility and manufacturing scalability. Autologous cells avoid donor–recipient HLA mismatch, whereas syngeneic cells originate from a genetically identical donor. Autologous production nevertheless requires patient-specific manufacture and release, may be limited by starting-material quality, and can delay treatment. Allogeneic platforms can support banked starting material and batch manufacture, but introduce recipient rejection and, for donor T-cell products, graft-versus-host disease; conditioning or immunosuppression may also be required [2,5,6,7,8,9,10].

Both approaches have clinical precedents in regenerative therapy: autologous iPSC-derived dopaminergic progenitors have been reported in a single patient, and allogeneic iPSC-derived dopaminergic progenitors have been investigated in a phase I/II trial [11,12]. Autologous origin also does not ensure tolerance to introduced sequences, as illustrated by clinical anti-CAR T-cell responses [13]. Accordingly, the present review addresses the immune barriers accompanying the potential supply advantages of allogeneic products. “Off-the-shelf” denotes a manufacturing and availability model; compatibility with every recipient remains an unproven objective.

### 1.1. The Problem of Immune Barriers in Allogeneic Cell Therapy: Lessons from Allograft Rejection Biology

**Figure 1:**
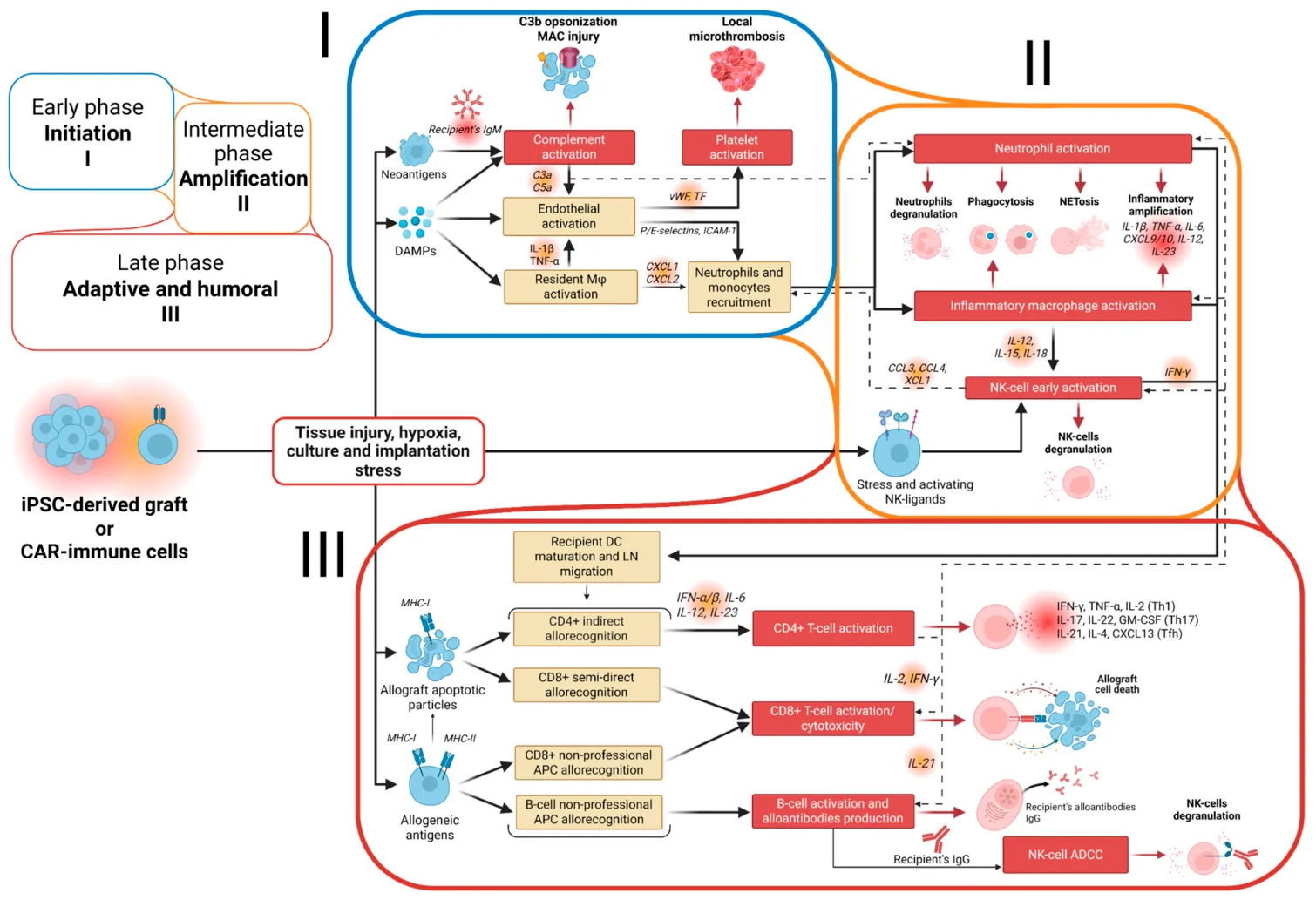
Interconnected phases of immune rejection of allogeneic cellular products. The schematic groups rejection-associated pathways into early initiation ((**I**); blue outline, upper center), intermediate inflammatory amplification ((**II**); orange outline, upper right), and adaptive and humoral responses ((**III**); red outline, lower region). Tissue injury, hypoxia, and culture or implantation stress generate damage-associated molecular patterns (DAMPs) and expose altered self-antigens. Early pathways include natural-antibody-associated complement activation, endothelial and resident-macrophage activation, platelet responses, microthrombosis, and leukocyte recruitment. Neutrophils, inflammatory macrophages, and natural killer (NK) cells then amplify tissue injury through inflammatory mediators and effector activity [14,15,16,17,18,19,20,21,22,23,24,25,26]. In the lower region, recipient antigen-presenting cells (APCs) acquire donor material. Indirect CD4^+^ T-cell recognition involves donor-derived peptides presented by recipient MHC-II; semi-direct recognition involves intact donor MHC–peptide complexes acquired and displayed by recipient APCs. Effector CD8^+^ T cells can also directly recognize donor MHC-I on graft cells. B cells recognize native alloantigens, and CD4^+^ T-cell help supports alloantibody production. Antibodies can recruit complement and NK-cell antibody-dependent cellular cytotoxicity (ADCC), reinforcing earlier injury pathways [17,27,28,29,30]. Solid arrows show pathway relationships and effector outcomes; dashed connections indicate cross-talk and feedback. The phases overlap and do not define fixed intervals or mutually exclusive mechanisms. Their relative importance depends on the product, administration route, tissue environment, and recipient immunity. CAR, chimeric antigen receptor; DC, dendritic cell; iPSC, induced pluripotent stem cell; LN, lymph node; MAC, membrane attack complex; MHC, major histocompatibility complex; NETosis, neutrophil extracellular trap formation.

Allorejection involves a coordinated interaction between innate and adaptive immunity. Much of our mechanistic understanding comes from vascularized-organ transplantation, but the same major effector pathways also act against allogeneic cellular products. Their relative importance depends on the cell type, route of administration, and tissue environment. The main point is that rejection develops as a network of interacting mechanisms rather than through a single dominant pathway (Figure 1).

Immediately after administration or implantation, tissue manipulation, ischemic injury, and cellular stress can release damage-associated molecular patterns (DAMPs), including HMGB1, ATP, and cell-free DNA. These signals activate pattern-recognition receptors on recipient endothelium and phagocytes and promote NF-κB-dependent production of inflammatory cytokines such as IL-1β, IL-6, and TNF [14,15]. In blood-exposed grafts, natural antibodies and complement add another early layer of injury. C3a and C5a promote leukocyte recruitment and endothelial activation, whereas C3b deposition opsonizes donor cells and facilitates their clearance [16,17]. Complement, coagulation, platelets, and inflammatory cells can further reinforce one another at the graft–blood interface, producing thromboinflammatory damage before adaptive immunity is fully established [18,19].

Neutrophils and monocytes are recruited early to sites of tissue injury and contribute to inflammatory amplification. Monocytes can subsequently differentiate into macrophages and monocyte-derived dendritic cells, linking early tissue injury to antigen presentation and adaptive immune activation [20,21].

Natural killer (NK) cells provide another layer of surveillance. They can recognize donor cells that lack appropriate HLA class I ligands for inhibitory NK receptors. This becomes particularly important when HLA expression is deliberately reduced to limit T-cell recognition. Activated NK cells can kill donor cells directly and produce IFN-γ, while donor-specific antibodies can further recruit NK-cell activity through Fc receptors and antibody-dependent cellular cytotoxicity [22,23,24,25,26].

Adaptive immunity develops in parallel. Recipient dendritic cells acquire donor material and support indirect recognition of donor-derived peptides presented by recipient MHC molecules. Acquisition of intact donor MHC–peptide complexes permits semi-direct recognition, whereas direct recognition involves donor MHC displayed on donor cells. Effector CD8^+^ T cells mediate cytotoxicity, and CD4^+^ T cells provide help for CD8^+^ T-cell activation as well as B-cell activation and alloantibody production [27,28,29,30]. The resulting antibodies can activate complement and recruit innate effector cells, reinforcing the inflammatory network that initiated graft injury [17].

These mechanisms can be viewed as three overlapping phases. Early initiation is dominated by tissue injury, DAMP sensing, complement, coagulation, platelets, and early myeloid responses. This is followed by inflammatory amplification involving neutrophils, monocytes/macrophages, NK cells, cytokines, and chemokines. Adaptive and humoral responses then become increasingly important through T-cell recognition, B-cell activation, donor-specific antibodies, and antibody-supported effector mechanisms. The boundaries between these phases are not strict, and several pathways remain active throughout the response.

The relative contribution of each mechanism also differs between products. Intravascular or intraportal products immediately encounter antibodies, complement, coagulation factors, platelets, and circulating myeloid cells. Locally implanted grafts may instead be affected more strongly by tissue injury, hypoxia, delayed vascularization, resident immune cells, and local inflammatory gradients [5,7,18,19]. This diversity helps explain why control of a single immune pathway rarely provides durable protection.

### 1.2. Limits of Current Hypoimmune Engineering Strategies

A central adaptive barrier in allotransplantation is recognition of donor HLA by recipient T cells. Direct allorecognition allows recipient T-cell receptors to recognize intact foreign HLA molecules on donor cells. In indirect allorecognition, recipient antigen-presenting cells process donor-derived material and present it to CD4^+^ T cells, supporting chronic alloimmunity and B-cell responses [29,30,31]. Allospecific antibodies add another route of injury through complement and Fc-receptor-dependent effector mechanisms [17].

Innate immunity creates additional barriers. NK cells can eliminate graft cells with insufficient self-HLA signals through missing-self recognition, which becomes especially relevant when HLA is reduced to evade T cells [25,26,32]. Stress-induced activating ligands can further increase NK-cell cytotoxicity. Macrophages also contribute to clearance, particularly when inhibitory signals such as CD47 are insufficient or are overridden by inflammation [33].

Several genetic strategies have therefore been developed to create hypoimmune donor cells [7,8,34]. One approach is an “HLA cloak”. B2M disruption reduces surface HLA class I and CD8^+^ T-cell recognition [35]. To limit the resulting NK-cell susceptibility, HLA-E can be introduced to engage inhibitory CD94/NKG2A signaling, or HLA-A and HLA-B can be selectively removed while HLA-C is preserved [36,37]. More complex platforms combine B2M and CIITA disruption with additional inhibitory signals such as CD47, thereby addressing T cells, NK cells, and phagocytes at the same time [38,39]. Hypoimmune iPSC derivatives have now shown long-term survival in immunocompetent allogeneic rhesus macaques [40].

A complementary “immune cloak” uses inhibitory surface molecules or local tolerogenic signals. PD-L1, HLA-G, CD47, and locally produced immunomodulatory cytokines have been used to reduce T-cell, NK-cell, or macrophage responses and to promote a more tolerogenic graft environment [33,38,41]. Clinical feasibility is also beginning to emerge: immune-edited allogeneic primary islets have been transplanted into a patient with type 1 diabetes without systemic immunosuppression [42].

Current stealth engineering is therefore already multilayered. Recent work combines HLA modulation with NK inhibition, phagocytosis control, altered immune-cell contact, antibody protection, and local immune regulation [8,43]. The remaining problem is that these approaches do not cover all parts of the rejection response equally well.

Reducing direct T-cell recognition does not prevent early inflammatory or blood-contact injury, complement deposition, macrophage or neutrophil attack, inflammatory cytokine toxicity, or terminal cell-killing pathways. Strong HLA suppression can also shift immune pressure toward NK cells, while checkpoint-based protection may change under inflammatory conditions [33,44].

Immune escape also does not guarantee product survival or function. Islet transplantation shows that IBMIR-like inflammation, hypoxia, delayed revascularization, oxidative stress, and cytokine injury can remove a substantial fraction of a graft before stable adaptive rejection is established [18,19]. IFN-γ and other inflammatory signals can increase HLA and antigen-processing programs, while hostile tumor or ischemic environments can impair therapeutic cells independently of alloantigen recognition [45,46].

Similar limitations are seen in allogeneic CAR products. TCR disruption reduces graft-versus-host liability but may also impair persistence, and FAS-dependent killing remains a route of host-mediated elimination even after conventional allogeneic engineering [47,48]. Antibodies against donor or engineered antigens provide another residual pressure. CD64 overexpression can reduce antibody-mediated killing, but it does not prevent formation of the antibodies themselves [44].

The required solution therefore depends on the product. CAR-based cells must evade rejection while retaining activation, immune-synapse formation, trafficking, proliferation, cytokine responsiveness, antitumor activity, and rapid clinical control. iPSC-derived regenerative grafts instead require long-term tissue integration, lineage-specific function, inflammatory resilience, vascular compatibility, balanced T- and NK-cell protection, and continued eliminability of abnormal cells [8]. Current engineering addresses several of these barriers, but it does not yet provide uniform protection across the full rejection response.

### 1.3. Poxviruses: A Model and Source of Translational Immune-Evasion Principles

**Figure 2:**
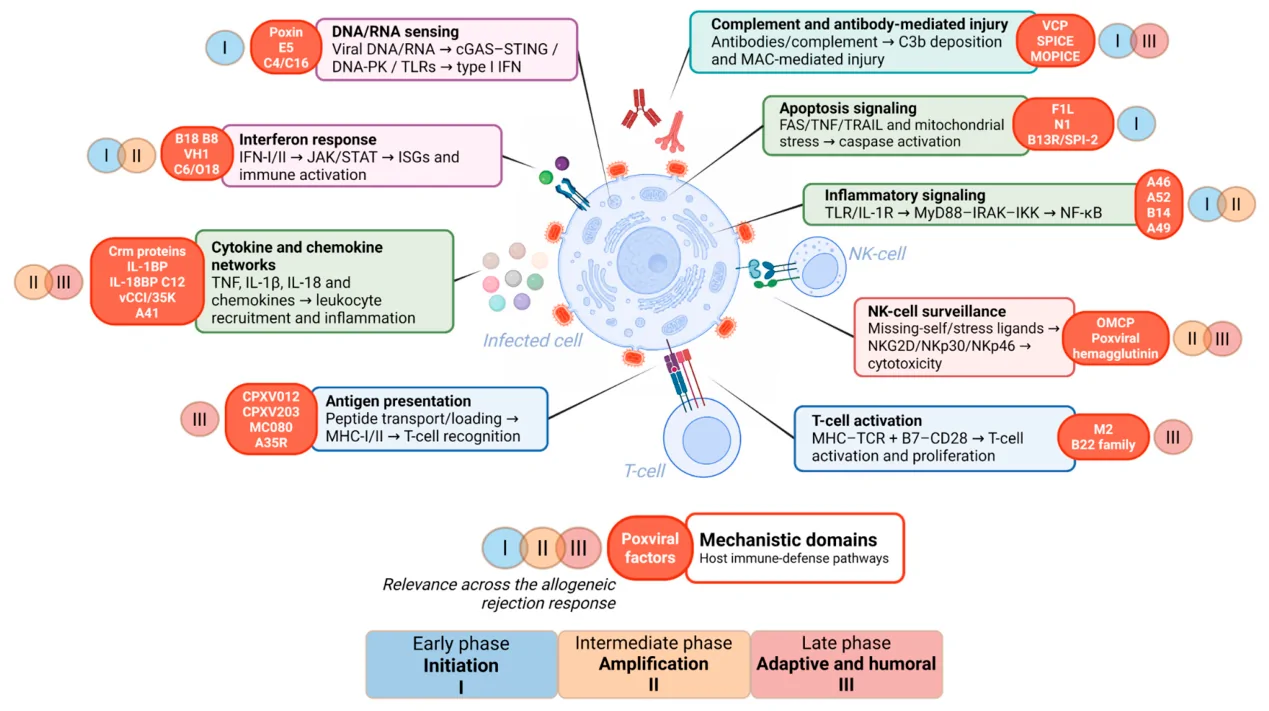
Poxviral immunomodulatory mechanisms in relation to the phases of allogeneic rejection. Representative host-defense pathways are arranged around an infected cell, with corresponding poxviral factors identified in red/orange boxes. The domains encompass nucleic-acid sensing, interferon responses, cytokine and chemokine networks, antigen presentation, T-cell activation, NK-cell surveillance, inflammatory signaling, complement-associated injury, and apoptosis. Circular markers map these domains onto initiation (I; blue), amplification (II; orange), and adaptive/humoral responses (III; muted red), as named in the lower legend. Overlapping markers indicate relevance to more than one phase; colors outlining the pathway boxes group mechanistic domains rather than rejection phases. The assignments are conceptual and non-exclusive. The infected cell illustrates the biological context in which the mechanisms were characterized; it does not represent infection of a therapeutic product or establish efficacy after transfer into that product. NK-receptor modulation can have receptor-specific activating as well as inhibitory effects [49,50,51,58,59]. IFN, interferon; ISGs, interferon-stimulated genes; NK, natural killer; MAC, membrane attack complex. Arrows within pathway boxes indicate the sequence or direction of the stated processes; connecting lines associate each mechanistic domain with the illustrated cellular context.

The focus on poxviruses follows from the biological scope of allogeneic cell rejection. Durable graft function is threatened by both immune recognition and the inflammatory injury that can precede or accompany adaptive rejection. The poxvirus literature encompasses intracellular immune regulation, extracellular cytokine and chemokine antagonism, complement control, and modulation of cellular injury within an extensively characterized viral family [49,50,51,52]. This repertoire provides a coherent basis for examining the initiation, amplification, and adaptive/humoral phases described in Section 1.1 and Figure 2, including pressures that persist when donor HLA-dependent recognition is reduced.

The resulting analytical value is the ability to distinguish control of antigenic recognition from protection against downstream injury, and cell-intrinsic effects from changes in the surrounding tissue environment. These distinctions are relevant to the different requirements of circulating CAR cells and implanted regenerative grafts. Poxviral mechanisms thus broaden the review beyond antigen-presentation control and provide a basis for evaluating which conclusions from infection biology have independent support in transplantation.

Such support exists for selected mechanisms in the wider poxvirus family. Studies using noninfectious recombinant proteins have reported reduced transplant vasculopathy or improved graft outcomes in rodent allograft models [53,54,55]. A recombinant myxoma-virus-derived serpin, Serp-1, was also evaluated in a randomized phase II pilot trial involving 48 patients undergoing coronary intervention; key safety outcomes did not differ from placebo [56]. These findings demonstrate that some poxviral activities can be studied outside productive infection. Their evidential scope remains limited: organ-transplant experiments and short-course recombinant-protein exposure do not establish the safety or effectiveness of sustained expression in CAR or iPSC-derived products.

Evidence from other viral families further supports the broader relevance of viral immune regulation [57]. The poxvirus focus therefore adds a systematic analysis of recognition, inflammation, and graft injury to this developing literature. This thematic focus does not establish superior efficacy, lower immunogenicity, or greater safety relative to approaches derived from other viral families. Such conclusions would require direct comparisons in the same therapeutic cell type under comparable conditions.

### 1.4. Aim and Scope of the Review

This review examines how poxviral immune-evasion mechanisms may inform the development of low-immunogenic allogeneic CAR and iPSC-derived cell products. Mechanism-level Tier 1–3 assignments are made in Section 3, Section 4, Section 5 and Section 6 and the accompanying mechanism-specific tables according to the proximity of cell-therapy evidence, compatibility with product function, and control burden. Section 7 integrates these assessments into product-specific design architectures and evidence gates for allogeneic CAR products and iPSC-derived regenerative grafts. Section 8 addresses the cross-cutting boundaries of therapeutic-function preservation, excessive immune escape, independent eliminability, transgene exposure and immunogenicity, and final-product manufacturing evidence.

The aim is not to advocate the direct transfer of poxviral proteins into therapeutic cells. Instead, the review considers whether individual mechanisms are better suited to controlled transgene expression, structure-guided redesign, transient or locally restricted delivery, or modification of the corresponding host pathway. The central question is which poxviral principles can reduce rejection without compromising the function, safety, and clinical control of the cellular product.

## 2. Current Hypoimmune Engineering in CAR and iPSC-Derived Products

Current hypoimmune engineering extends beyond HLA deletion. Recent platforms combine antigen-presentation control with NK-cell inhibition, phagocytosis checkpoints, antibody protection, altered immune-cell contact, or local immune regulation [43]. However, the evidence comes from different intervention formats, including stable final-product engineering, transient surface modification, biomaterials, recipient-directed treatment, and mechanistic models. These approaches should therefore be interpreted according to the specific mechanism and experimental context tested.

Product requirements also differ. Allogeneic CAR-T and CAR-NK cells must retain expansion, trafficking, immune-synapse formation, cytokine responsiveness, cytotoxicity, and clinical controllability. iPSC-derived regenerative grafts instead require stable differentiation, tissue-specific function, integration, and continued eliminability of abnormal cells [60,61,62]. Thus, persistence should be interpreted together with product-specific function and the conditions under which it was measured.

### 2.1. Hypoimmune Engineering in Allogeneic CAR Products

Allogeneic CAR products face three distinct problems: donor-to-host alloreactivity, recipient rejection of the therapeutic cells, and immune responses against CAR-derived or other engineered sequences [63,64,65].

Clinical allogeneic CAR-T platforms such as UCART19, TT52CAR19, ALLO-715, and BE-CAR7 show that donor-TCR disruption can support off-the-shelf treatment when combined with lymphodepletion [9,10,64,66]. These studies establish clinical feasibility, but persistence cannot be attributed to cell-intrinsic immune evasion alone because conditioning and subsequent immune recovery strongly affect CAR-cell kinetics. TCR disruption should therefore be viewed primarily as control of graft-versus-host disease (GVHD), not as protection from recipient immunity; it may also influence CAR-cell persistence [47].

Protection from host immunity requires coordinated control of T- and NK-cell recognition. Combined B2M and CIITA disruption reduces HLA-I- and HLA-II-dependent recognition, but loss of HLA-I also removes inhibitory signals for NK cells [65]. HLA-E-based constructs and non-HLA inhibitory approaches such as CD300a or LLT1/CD161 signaling can restore selected inhibitory inputs [67,68,69]. Their performance varies with recipient NK-cell phenotype, so HLA reduction and NK protection should be treated as one coupled design problem rather than as independent edits.

Multilayer CAR platforms further show that HLA control alone is insufficient. HIP CAR-T cells combine TCR and HLA editing with CD47-mediated protection, whereas viral comparators such as HHV-8 K3/K5, EBV BNLF2a, and HIV-1 Nef reduce antigen presentation without reproducing complete HLA deletion [57,63,70,71,72]. BNLF2a-based engineering also reduced anti-CAR T-cell recognition. These approaches address direct recognition, but recipient APCs can still acquire product-derived material, and antibodies against engineered antigens may still develop.

Non-HLA mechanisms address additional routes of clearance. CD47 reduces macrophage-mediated elimination, CD64 limits IgG-dependent ADCC and antibody-triggered complement injury, and CD54/CD58 editing reduces productive immune-cell contact [33,44,73]. FAS disruption and engineered SERPINB9 further show that resistance to terminal killing can improve survival of allogeneic CAR cells after recognition has occurred [48,74]. Such protection must, however, preserve CAR-target synapse formation, cytotoxicity, and an independent means of cell elimination.

Finally, allogeneic CAR products remain living drugs. Editing completeness, genome integrity, clonal composition, expansion, trafficking, repeated killing, and safety-switch function are therefore part of immune-product quality rather than separate manufacturing issues [75,76,77,78]. CAR-NK cells avoid the donor-TCR/GVHD problem but retain host-rejection and persistence constraints [60].

### 2.2. Hypoimmune Engineering in iPSC-Derived Products

For regenerative grafts, immune engineering must reduce direct T-cell recognition, prevent compensatory innate attack, preserve the differentiated lineage, and retain a way to eliminate abnormal cells.

Selective HLA deletion, HLA-E expression, and broader hypoimmune architectures have reduced T- and NK-cell recognition in stem-cell-derived products and supported prolonged survival in preclinical models [36,40,79]. Edited primary allogeneic islets have also shown early clinical survival without systemic immunosuppression in one recipient [42]. These results provide strong proof of principle for direct recognition control, but they do not eliminate indirect or semi-direct allorecognition, recipient variability, or disease-specific autoimmunity [29,80].

HLA control can be complemented by checkpoint, phagocytosis, and adhesion engineering. Synthetic checkpoint engagers, CD24 expression, ICAM-1 disruption, and multicomponent immune cloaks have reduced NK-, macrophage-, or contact-dependent injury in differentiated endothelial, cardiovascular, and neural products [61,62,81,82]. These studies also show why immune phenotypes cannot be assumed to transfer unchanged between lineages.

Antibody, complement, and blood-contact injury can also be targeted. CD55 and CD64 reduce selected complement- and IgG-dependent effector mechanisms, while transient surface regulators and anticoagulant strategies can reduce early blood-interface injury [44,83,84,85,86]. Much of this evidence comes from islets or other blood-contact models and therefore should not be generalized to locally implanted grafts. These interventions also act downstream of antibody formation and do not prevent recipient B-cell priming or immune memory.

Inflammatory pathways provide another layer of engineering. TYK2 perturbation, CXCL10 deletion, and A20-mediated threshold control demonstrate that IFN-, chemokine-, and NF-κB-linked mechanisms can be modified in stem-cell-derived or graft-relevant systems [87,88,89]. Local cytokine circuits and CXCL12-containing biomaterials provide additional spatially restricted protection [41,90]. By contrast, protection from neutrophil- and NET-associated injury remains less developed; current evidence mainly establishes that this residual barrier exists and can be modified in selected experimental systems [91].

The relevant unit for both efficacy and safety is the final differentiated graft rather than the parental iPSC clone. Differentiation and inflammatory exposure can alter HLA expression, innate-regulatory molecules, tissue function, and immune-cell interactions [62,82,92]. Final-product composition is equally important: residual progenitors, non-target lineages, incomplete editing, and phenotype drift can affect both immune recognition and safety. Platforms that combine immune protection with independent elimination provide an important design precedent [93,94,95,96].

### 2.3. Remaining Gaps: Mechanism-Level Coverage and Stress-Tested Functional Immunogenicity

#### 2.3.1. Asymmetric Coverage Across the Three Rejection Phases

Current engineering reaches mechanisms in all three rejection phases, but with different depths and levels of evidence (Table 1). Early-phase strategies mainly address individual blood- or tissue-interface mechanisms; intermediate-phase approaches target selected NK, myeloid, inflammatory, contact, or killing pathways; and late-phase engineering is most developed for direct HLA-dependent recognition. Important gaps remain in integrated blood-interface control, coordinated inflammatory responses, recipient-APC priming, immune memory, and de novo humoral immunity.

The phases should therefore be used as an organizational map rather than classified as solved or unsolved. Most current strategies control a defined component of rejection rather than an entire phase.

#### 2.3.2. Product-Matched Functional Validation and Evidence Boundaries

Experimental systems also support different conclusions. Human cocultures test direct cellular recognition but not long-term tissue integration [68]. Humanized models add in vivo context but may incompletely reproduce clinically relevant human immune responses [95], whereas xenogeneic models may introduce species-specific effects [91]. Immunodeficient recipients are useful for testing engraftment and product fitness but cannot establish resistance to an intact allogeneic immune system [97]. Likewise, transient surface modification, biomaterials, and recipient-directed pharmacology demonstrate pathway tractability without creating a stable cell-intrinsic phenotype [84,86,90,98].

For both CAR and iPSC-derived products, immune protection should therefore be tested in the final therapeutic cell under product-matched stress and together with preserved therapeutic function and independent eliminability [71,92,94,99,100]. Table 1 summarizes the current coverage, residual gaps, and the evidence boundaries that should guide interpretation.

**Table 1:** Current engineering coverage, evidence context, and unresolved gaps in allogeneic CAR and iPSC-derived cell products.

Product/Barrier	Representative Strategies and Evidence Context	Demonstrated Coverage	Main Residual Gap or Validation Requirement
CAR—donor TCR/GVHD liability; parallel to host-versus-product rejection	TRAC/TRBC and CD52 editing in clinical allogeneic CAR platforms under lymphodepletion [9,10,64,66]	Reduces donor-TCR-mediated alloreactivity and enables off-the-shelf administration	Does not protect against recipient immunity; persistence remains influenced by conditioning, immune recovery, and residual unedited cells
CAR—direct HLA recognition, NK counter-pressure, and anti-transgene T cells; Phases II–III	B2M/CIITA editing with HLA-E or non-HLA NK-inhibitory modules; HIP and viral antigen-presentation comparators [57,63,65,67,68]	Reduces direct HLA-dependent and selected anti-CAR T-cell recognition while restoring selected NK-inhibitory signals	Recipient NK diversity, indirect presentation, anti-product antibodies, HLA-replete subpopulations, and abnormal-cell surveillance remain
CAR—macrophage, antibody, contact, and terminal-killing mechanisms; Phases I–III	CD47, CD64, CD54/CD58 editing, FAS disruption, and SERPINB9 engineering [33,44,48,73,74]	Reduces selected phagocytic, IgG-dependent, contact-dependent, or death-mediated clearance	Does not prevent upstream priming; CAR-target synapse, repeated killing, and independent eliminability must be preserved
CAR—manufacturing completeness and living-drug control; cross-phase	Purification, multiplex/base editing, genome-integrity analysis, and longitudinal clonal monitoring [75,76,77,78]	Reduces residual immunogenic populations and some editing-related liabilities	Final assessment must integrate editing completeness, genome integrity, potency, trafficking, persistence, and safety-switch competence
iPSC graft—direct HLA recognition and NK missing-self; Phases II–III	Selective HLA editing, HLA-E, and hypoimmune architectures in differentiated products and allogeneic models [36,40,42,79]	Strongest current evidence for coordinated reduction in direct T- and NK-cell recognition	Indirect/semi-direct presentation, recurrent autoimmunity, recipient diversity, and long-term surveillance remain
iPSC graft—checkpoint, macrophage, and adhesion-mediated injury; Phase II	Checkpoint engagers, CD24, ICAM-1 editing, and lineage-specific immune cloaks [61,62,81,82]	Reduces selected innate clearance and productive immune-cell contact	Durability under inflammation, transferability between lineages, and effects on normal tissue interactions require final-product testing
iPSC graft—antibody, complement, coagulation, and blood-contact injury; Phases I and III	CD55/CD64, APT070, tissue-factor control, and thrombomodulin-based approaches in human or graft-relevant models [44,83,84,85,86]	Demonstrates control of selected complement-, IgG-, coagulation-, or phagocytosis-related components	No human iPSC final product provides integrated control of complement, coagulation, platelets, endothelium, and PMN/NET injury; de novo antibody formation remains
iPSC graft—inflammatory and granulocyte amplification; Phases I–II	TYK2, CXCL10, A20, local cytokine/CXCL12 systems, and PMN-oriented approaches [87,88,89,90,91]	Shows that selected inflammatory, recruitment, and granulocyte-related pathways are tractable	Coordinated inflammatory-state control and direct human-allogeneic final-product protection from PMN/NET injury remain limited
iPSC graft—final-product composition and independent control; cross-phase	Safety switches, final-lineage enrichment, and spatial/compositional analysis [93,94,95,96]	Links immune protection to differentiated-product identity and independent elimination	Residual progenitors, non-target lineages, incomplete editing, phenotype drift, and long-term switch competence remain
Cross-product—recipient APC priming, memory, and de novo humoral immunity; Phase III	Recipient-APC uptake/cross-dressing and recipient-directed tolerance studies [29,101,102]	Shows that these mechanisms remain active after donor-cell HLA attenuation	No general product-intrinsic strategy prevents indirect presentation, memory T-cell recall, B-cell priming, or new antibody formation
Cross-product—evidence and model boundary	Human cocultures, humanized models, xenogeneic models, immunodeficient transplantation, transient coatings, biomaterials, and recipient pharmacology [68,84,91,95,97,98]	Each model resolves a specific part of immune protection or transplantation fitness	Claims should be restricted to the tested format; final differentiated products require product-matched immune stress, preserved function, and independent eliminability

## 3. Poxviral Modulation of Interferon Responses

Interferons link innate sensing to antigen presentation, inflammatory recruitment, antiviral defense, and immune surveillance. Their effect on an allogeneic cell product is not one-directional. IFN-γ can increase both HLA molecules and inhibitory ligands in differentiated iPSC products, while type I IFN can reshape the β-cell immunopeptidome and increase inflammatory stress [103,104,105].

Type I and type II IFNs also use different signaling routes. Type I IFNs signal through IFNAR1/2 and TYK2/JAK1 to activate STAT1–STAT2–IRF9/ISGF3. IFN-γ signals through IFNGR1/2 and JAK1/JAK2, with STAT1 as the main transcriptional effector [106,107,108,109]. This distinction matters when IFN signaling is modified in the therapeutic cell, the target tissue, or neighboring host cells.

**Figure 3:**
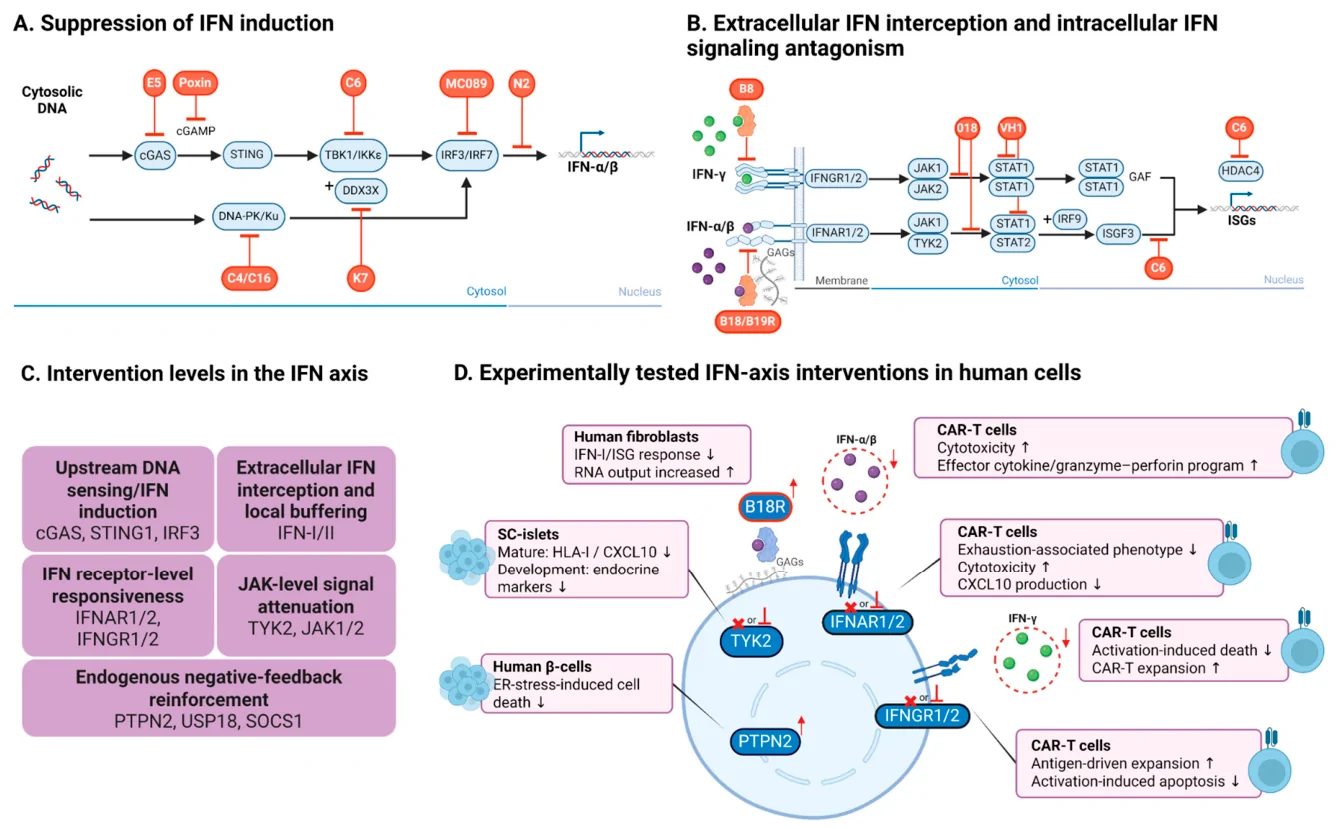
Interferon regulation by poxviruses and related evidence in human cells. (**A**) Representative poxviral effects on cytosolic sensing and downstream type I interferon (IFN) induction. (**B**) Extracellular IFN interception and intracellular antagonism of IFN-receptor signaling. (**C**) Host-pathway levels corresponding to these mechanisms, from sensing and extracellular interception to receptor responses, signal transmission, and endogenous feedback. (**D**) Findings from published human-cell studies, with the cell model and direction of the reported outcome identified in each callout. The examples span fibroblasts, CAR-T cells, stem-cell-derived islets, and human β cells; differences between mature-cell responses and developmental effects are shown where relevant. These findings illustrate context dependence and do not establish that the depicted changes are equivalent or jointly beneficial. Red T-bars denote inhibition or blockade; red crosses, gene disruption; upward and downward arrows, increased and decreased expression or reported outcomes, respectively. Host components are blue/grey, poxviral factors red/orange, type I IFN purple, and IFN-γ green. GAGs, glycosaminoglycans; ISGs, interferon-stimulated genes; SC-islets, stem-cell-derived islets.

Poxviruses show that the IFN response can be controlled at several levels (Figure 3). They suppress IFN induction, intercept extracellular IFNs, and inhibit intracellular signaling [110,111,112,113,114]. These mechanisms are useful because they separate intervention levels that would otherwise be grouped under the broad term “IFN suppression.” Human perturbation studies then show which of these levels can be modified without assuming that complete IFN resistance is desirable.

### 3.1. Poxviral Control Points Across the IFN Axis

Poxviruses can suppress the pathway before IFN is produced. Poxin degrades the cGAS product 2′3′-cGAMP, E5 antagonizes cGAS, and C4/C16 interfere with DNA-PK/Ku-dependent sensing [115,116,117]. C6, K7, and N2 act further downstream through the TBK1/IKKε–IRF3/IRF7 axis [110,118,119]. The main lesson is that IFN induction can be interrupted at several molecular depths.

These upstream nodes are poor candidates for broad permanent suppression in therapeutic cells. DNA-PK/Ku also supports DNA double-strand-break repair, and human TBK1 deficiency can increase inflammatory cell death rather than simply reduce IFN output [120,121,122]. Poxviral DNA-sensing antagonists are therefore more useful for identifying pathway vulnerabilities and safety boundaries than as direct templates for stable editing.

Extracellular interception provides a more restricted form of control. B18/B19R binds type I IFNs before they reach IFNAR and can remain associated with cell surfaces through glycosaminoglycans [111,123,124]. Importantly, B18R remains active outside productive infection. Recombinant B18R and B18R mRNA have been used to suppress type I IFN responses during RNA delivery and human-cell reprogramming [125,126,127]. This makes B18/B19R a useful model for local or transient cytokine buffering.

B8-like proteins apply the same extracellular principle to IFN-γ, but the consequences are different. B8 binds IFN-γ and shows marked species dependence [112,128]. A diffusible IFN-γ trap would act on several cell populations at once. This is important in CAR therapy because IFNγR signaling in solid-tumor target cells can support productive CAR–target interaction [129].

Poxviruses also act after IFN reaches its receptor. VACV 018 prevents productive STAT1 recruitment to activated IFN receptors, VH1 dephosphorylates STAT1 Tyr701, and C6 limits type I IFN transcription through STAT2- and HDAC4-dependent nuclear mechanisms [113,114,130,131]. These mechanisms separate receptor coupling, STAT activation, and nuclear transcription. They do not provide a rationale for constitutive blockade of the entire JAK/STAT pathway.

Poxviruses also counter the downstream antiviral state. E3, K3, D9/D10, and C7/K1 interfere with pathways involved in translational arrest, RNA degradation, necroptosis, and host restriction [132,133,134,135,136]. These mechanisms are not treated as preferred intervention levels in Figure 3C. Instead, they identify antiviral and stress-defense functions that should remain intact when upstream IFN signaling is attenuated.

### 3.2. Human Interventions Show That IFN Tuning Is Context Dependent

The host-side intervention space in Figure 3C is deliberately narrower than the full poxviral repertoire. Human studies provide the clearest evidence for extracellular buffering, receptor-level modulation, TYK2 attenuation, and endogenous negative feedback.

Human β cells and stem-cell-derived endocrine cells provide strong evidence for TYK2 as a tunable node. Reducing TYK2 activity lowers IFN-α-induced HLA-I and inflammatory programs, but complete TYK2 loss can also affect endocrine development [87,137,138]. These findings favor controlled or reversible attenuation over a general TYK2 knockout strategy.

CAR-T studies show a different pattern. Persistent cell-intrinsic type I IFN signaling can reinforce dysfunction during chronic antigen stimulation, whereas low-strength IFN-I exposure restricted to ex vivo manufacturing can improve subsequent CAR-T activity [139,140]. In chronically stimulated human CAR-T cells, antibody-mediated IFNAR blockade reduced the exhaustion-associated phenotype and improved cytotoxicity in control cells, while providing little additional benefit after EGR2 deletion, consistent with both interventions converging on the same inhibitory IFN-I program [140]. Infection-associated combination models provide a complementary caution: IFNAR1-deficient CAR cells can resist high local IFN-I but become more susceptible to NK-cell attack outside lymphodepletion [141]. Thus, type I IFN control depends on signal strength, exposure duration, and the immune context rather than on a simple IFNAR-on versus IFNAR-off state.

A similar compartment effect is seen with IFN-γ. At the ligand level, neutralizing IFN-γ or deleting IFNG in human CAR-T cells preserved cytotoxicity against hematologic targets while increasing CAR-T proliferation and reducing IFN-γ-dependent macrophage activation [142]. At the responder-cell level, IFNGR1 disruption in CD28-costimulated CAR-T cells reduces IFN-γ-induced apoptosis and can improve expansion and persistence [143]. The opposite requirement is seen in the target compartment: intact IFNγR signaling in solid-tumor cells can support productive CAR–target adhesion and killing [129]. Ligand neutralization, CAR-cell receptor attenuation, and target-cell receptor loss are therefore not interchangeable interventions. This argues against constitutive B8-like IFN-γ trapping across the whole tissue.

Endogenous negative-feedback regulators require the opposite interpretation from receptors or positive JAK/STAT components: strengthening the brake can attenuate IFN output, whereas loss of the brake generally increases signaling. Human gain- and loss-of-function studies support this bidirectionality. SOCS1 or SOCS3 overexpression suppresses IFN-α- and IFN-γ-induced STAT1 phosphorylation and downstream transcription in human cells, whereas SOCS depletion increases IFN responsiveness [144]. Human pluripotent cells provide a related endogenous example: high SOCS1 expression is associated with weak IFN-β responsiveness, and SOCS1 knockdown increases STAT phosphorylation and ISG induction [145].

PTPN2 further shows why the desired direction is product-dependent. PTPN2 deficiency increases inflammatory signaling and impairs differentiation and functional maturation of human stem-cell-derived islets, whereas PTPN2 disruption or inhibition enhances expansion and several effector properties of human CAR-T cells [146,147]. Gain-of-function evidence provides the reciprocal mechanistic precedent: adenoviral PTPN2 overexpression in human β-cell models partially protected cells from ER-stress-induced death [148]. This supports reinforcement of endogenous feedback as a graft-oriented concept, but it does not yet demonstrate a stable low-immunogenic phenotype in a final iPSC-derived graft. Endogenous brakes should therefore be treated as product-specific rheostats rather than universal stealth targets.

### 3.3. Product-Specific Interpretation of IFN Modulation

For allogeneic CAR products, IFN control should be selective and reversible. Type I IFN can improve CAR-T cells when used briefly during manufacturing but can promote dysfunction when signaling becomes persistent. IFN-γ neutralization can also be compatible with CAR-T activity in hematologic models. However, tissue-wide IFN-γ trapping is harder to predict because tumor and stromal compartments may require intact IFN-γ signaling. Product-restricted receptor or signaling modulation can therefore be easier to control than constitutive secretion of a diffusible cytokine trap.

For iPSC-derived regenerative grafts, local attenuation is more attractive. Type I IFN/TYK2 signaling can increase antigen presentation, chemokine output, and inflammatory stress in human endocrine cells [87,103,137]. Yet this conclusion cannot simply be transferred to every iPSC-derived tissue. IFN-γ–JAK1 signaling has been linked to reduced stem-cell-derived RPE engraftment, while IFN-γ-treated iPSC-derived cardiomyocytes increased both HLA molecules and inhibitory ligands [104,105]. The final differentiated product must therefore be tested directly.

The same caution applies to endogenous feedback. Available gain-of-function evidence supports reinforcement of PTPN2- or SOCS-like brakes as a mechanistic route to limit inflammatory signaling or stress, but stable feedback enhancement has not yet been validated as a low-immunogenicity modification in a final differentiated iPSC-derived graft [144,148].

Overall, local B18-inspired buffering and reversible TYK2 attenuation are Tier 2 concepts. Feedback reinforcement can also be considered a Tier 2 concept when it is selective and product-specific, but broad constitutive reinforcement carries a higher control burden. Permanent suppression of upstream DNA sensing, broad JAK/STAT blockade, and loss of downstream antiviral defenses remain Tier 3. B8-like IFN-γ interception is strongly compartment dependent. The central design rule is simple: tune the IFN response at the level needed by the product rather than making the cell globally IFN-blind. Table 2 summarizes these mechanisms, the human evidence, and product-specific constraints.

## 4. Adaptive Immune Recognition: Antigen Presentation, Costimulation, and T-Cell Activation

Direct HLA-dependent recognition is one of the best-developed targets of hypoimmune cell engineering, but reducing donor HLA does not eliminate adaptive immunity. Recipient T cells can recognize intact donor HLA or donor-derived peptides, while recipient antigen-presenting cells (APCs) can continue to acquire donor material and support indirect or cross-dressed recognition [29]. Inflammatory exposure can further remodel antigen presentation in the final therapeutic lineage: type I interferons alter HLA-I expression and the immunopeptidome of human β cells, whereas IFN-γ can induce MHC-II-associated programs in differentiated graft-relevant cells [45,103].

**Figure 4:**
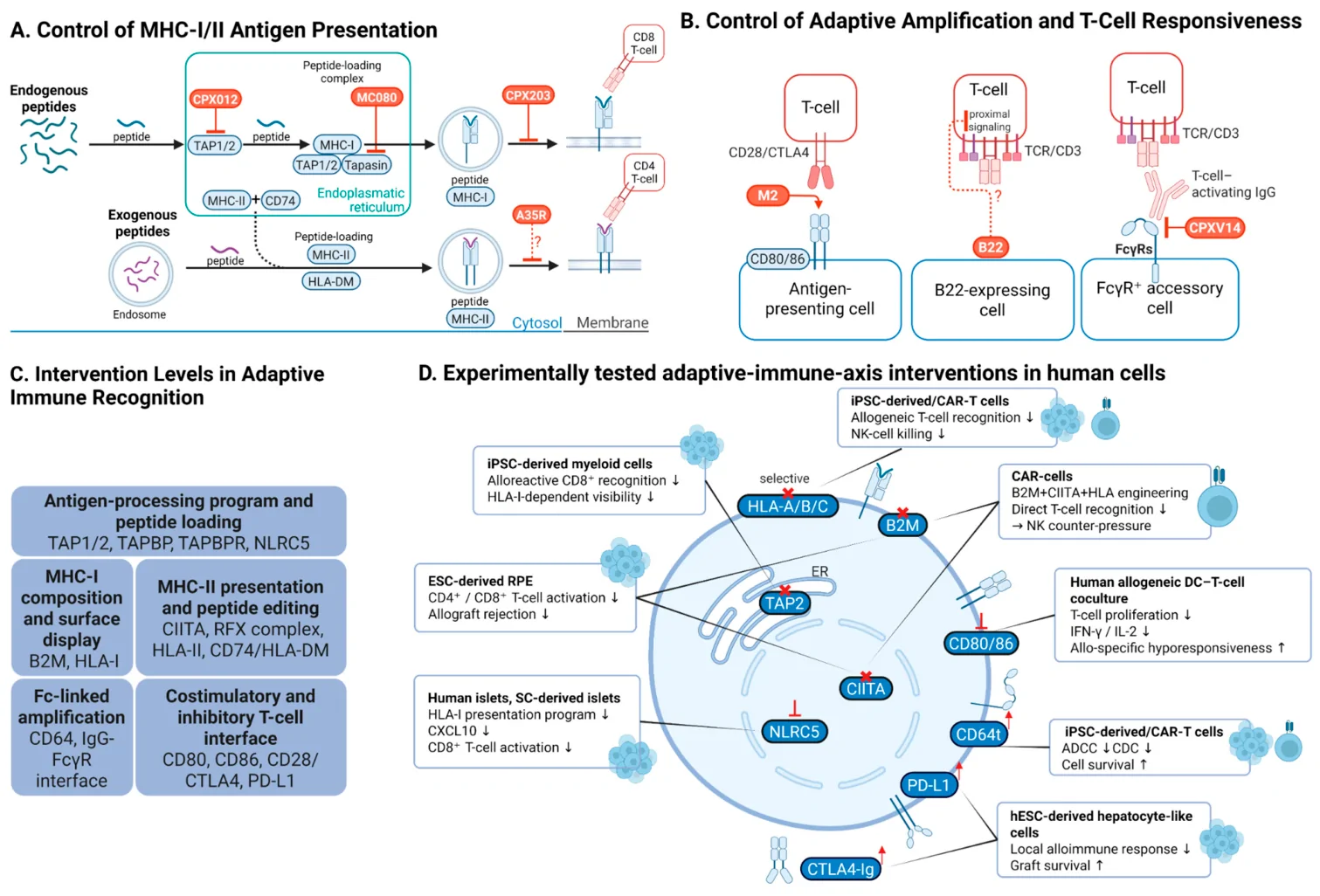
Poxviral effects on antigen presentation and T-cell activation in relation to human-cell evidence. (**A**) Representative effects on MHC-I peptide processing, loading, and trafficking, together with modulation of MHC-II-associated presentation. (**B**) Effects on costimulation, T-cell responsiveness, and antibody/Fc-receptor-linked accessory-cell activation. Dashed lines and question marks identify incompletely resolved steps. (**C**) Host-pathway levels relevant to adaptive recognition, including antigen processing, MHC display, costimulation, and Fc-linked amplification. (**D**) Published findings in the human-cell models specified in the callouts. Reduced direct T-cell recognition, antibody-dependent injury, and graft-associated responses are distinguished from the NK-cell pressure that can accompany altered MHC-I display. The panel juxtaposes separate studies and does not imply that every component was tested in the same product or that allogeneic rejection was eliminated. Red T-bars denote inhibition or blockade; red crosses, gene disruption; solid black arrows, pathway progression; upward and downward arrows, increases and decreases in expression or reported outcomes, respectively. Black leader lines link interventions to their study callouts. Host molecules are blue/grey and poxviral factors red/orange. ADCC, antibody-dependent cellular cytotoxicity; CDC, complement-dependent cytotoxicity; DC, dendritic cell; ESC, embryonic stem cell; RPE, retinal pigment epithelium.

Viral immune-evasion studies already show that antigen presentation can be reduced without complete HLA deletion. HHV-8 K3/K5, EBV BNLF2a combined with CIITA suppression, and controlled HIV-1 Nef expression have reduced host recognition of engineered human T cells or CAR-T cells through distinct forms of MHC-I or MHC-II modulation [57,63,70]. Poxviruses extend this principle by acting at several non-equivalent levels between peptide generation and T-cell activation. Figure 4 maps these viral interruption points onto the broader human intervention space.

### 4.1. Poxviral Control Points Across Adaptive Immune Recognition

Poxviruses separate MHC-I control into peptide supply, peptide loading, and surface trafficking. CPXV012 inhibits ATP-dependent peptide transport by TAP, whereas MC080 targets tapasin and destabilizes the peptide-loading complex. CPXV203 acts further downstream by retrieving mature MHC-I away from the cell surface [149,150,151,152]. CPXV012 and CPXV203 can therefore cooperate without acting through the same molecular mechanism [153].

The important trade-off is that these interventions change more than CD8^+^ T-cell visibility. TAP- and tapasin-dependent peptide supply contributes to HLA-E surface expression, and MC080 reduces both classical HLA-I and HLA-E. MC080 consequently suppresses antigen-specific CD8^+^ T-cell activation while producing donor-dependent changes in NK-cell recognition [154,155]. Potent inhibition of donor-cell MHC-I also does not abolish T-cell priming, because antigen can still reach recipient APCs through direct, indirect, or cross-priming routes [29,156,157].

Poxviral control extends beyond MHC-I. A35R reduces MHC-II-restricted antigen presentation and CD4^+^ T-cell activation, but its precise host target remains unresolved; CIITA and downstream MHC-II machinery should therefore be treated as host intervention nodes rather than molecular targets of A35R [158]. M2 instead acts extracellularly at the B7 interface. Recombinant oligomeric MPXV M2 binds human CD80/CD86 and suppresses B7-dependent activation of primary human T cells outside productive infection, whereas a monomeric variant retains binding but shows substantially weaker functional suppression [159]. This provides direct evidence that the M2 mechanism can operate autonomously at the human APC–T-cell interface.

CPXV14 adds an antibody-dependent amplification layer by engaging FcγRs on accessory cells and reducing antibody-supported T-cell activation [160]. B22-family proteins define a deeper boundary: they suppress human T-cell responses after receptor engagement, while the precise host receptor or molecular target responsible for this effect remains unresolved [161]. B22 therefore demonstrates that poxviruses can suppress the responder T cell itself, but it does not define a corresponding host-gene intervention domain.

### 4.2. Human Interventions Expand the Adaptive-Recognition Control Space

Human perturbation studies show that the antigen-processing pathway is experimentally tractable but cannot be treated as a set of interchangeable loss-of-function targets. TAP1 or TAPBP disruption in human embryonic stem cells reduced surface HLA-I and experimental immunogenicity while retaining pluripotency and differentiation capacity [162]. Targeted TAP2 disruption has also been used to generate human iPSCs and iPSC-derived myeloid cells with reduced recognition by pre-activated alloreactive CD8^+^ T cells [163]. The latter model is an antigen-presenting myeloid system rather than a regenerative graft, but it extends TAP-level tractability into human iPSC-derived cells.

The intervention space extends beyond TAP and tapasin. NLRC5 coordinates transcription of multiple MHC-I pathway genes and therefore represents a program-level regulatory node rather than a structural HLA target [164]. TAPBPR and endoplasmic-reticulum folding factors produce non-equivalent effects on HLA abundance and peptide repertoire, and PDIA3 disruption in human CD8^+^ and CAR-T cells enhanced effector activity rather than establishing a stealth phenotype [165,166,167]. Peptide trimming provides a further warning: combined ERAP1/ERAP2 loss can alter HLA-E peptide presentation and disrupt the inhibitory NKG2A–HLA-E checkpoint [168]. Surface HLA abundance alone is therefore insufficient to predict the immune consequence of antigen-processing edits.

MHC-II control is similarly multilayered. CIITA is the strongest engineered comparator, with direct evidence from CAR-T and hypoimmune hPSC platforms [38,39,63]. Downstream HLA-II expression, CD74-dependent intracellular trafficking, and HLA-DM-dependent peptide loading provide additional separable control points, several of which are inducible in human graft-relevant cells under inflammatory conditions [45]. These nodes expand the intervention map without implying that A35R directly targets them.

The T-cell activation threshold can also be shifted through positive or inhibitory surface interfaces. Clinical B7 blockade establishes the CD80/CD86–CD28/CTLA-4 axis as therapeutically tractable [169]. In a different intervention format, human ESCs engineered to express CTLA4-Ig together with PD-L1 generated differentiated grafts with localized protection from human alloimmune responses, whereas either module alone was insufficient in the reported system [170]. Lineage-restricted PD-L1 and CTLA4-Ig expression has subsequently been applied to hESC-derived hepatocyte-like cells to establish local immunomodulation after differentiation [171]. PD-L1 is not a molecular analogue of M2; these studies instead show that positive costimulation and inhibitory checkpoints provide distinct routes for regulating the broader T-cell activation interface.

The same distinction is required for Fc biology. Activating and inhibitory FcγRs operate in different recipient immune compartments and should not be treated as a uniform family of product-side knockout targets. Therapeutic-cell CD64 expression represents a different strategy based on IgG capture and has reduced antibody-dependent injury in CAR-T and iPSC-derived products [44]. The relevant intervention space is therefore the IgG–FcγR interface, not indiscriminate FCGR1/2/3 disruption.

### 4.3. Product-Specific Interpretation of Adaptive Immune Recognition

For allogeneic CAR products, the strongest logic is graded, cell-intrinsic control of antigen presentation while preserving the functions required of an active immune effector. BNLF2a, K3/K5, and Nef provide direct viral-comparator evidence that reduced antigen presentation can remain compatible with CAR activity [57,63,70]. However, peptide-processing or HLA attenuation must be interpreted together with the remaining NK-inhibitory architecture. Broad M2-like manipulation of the surrounding B7 interface is less contained, whereas B22-like responder T-cell suppression is poorly aligned with a product whose therapeutic function depends on intact activation and cytotoxicity.

For iPSC-derived regenerative grafts, stronger donor-cell HLA-I and HLA-II attenuation may be compatible with tissue-replacement function, but it still controls mainly direct recognition. Recipient APCs can continue to acquire donor material and sustain indirect CD4^+^ T-cell and humoral responses [29]. TAP-, tapasin-, or ERAP-level interventions also require particular caution when the graft depends on HLA-E-mediated NK inhibition because peptide-processing changes can alter both classical HLA-I presentation and the inhibitory HLA-E peptidome [155,168]. Local costimulatory or checkpoint modulation is more naturally aligned with a stationary graft than with a mobile immune effector, but the relevant interface and final differentiated lineage must be defined experimentally.

Taken together, the poxviral mechanisms reveal a broader candidate intervention space than the set of genes already used in current hypoimmune products. At the MHC-I level, peptide transport, peptide trimming, peptide loading, transcriptional coordination, and surface-HLA composition can be separated. MHC-II transcription, trafficking, and peptide loading are likewise distinct, while costimulatory, inhibitory-checkpoint, and IgG–FcγR interfaces provide additional levels beyond antigen presentation. These nodes are not equivalent engineering recommendations; Figure 4 distinguishes this candidate molecular space from the smaller set of interventions already tested in relevant human cells.

Applying the Tier 1–3 hierarchy, selective HLA and CIITA-based engineering and CD64-mediated antibody protection are closest to Tier 1. CPXV012-, CPXV203-, MC080-, A35R-, M2-, and CPXV14-derived principles remain Tier 2 because their poxviral mechanisms are well defined but product compatibility is supported mainly by host-pathway or parallel human evidence. B22 remains a Tier 3 boundary mechanism because its host target is unresolved and its responder-cell suppression does not provide a selective product-side stealth strategy [161]. The central design rule is therefore graded adaptive-visibility tuning with paired NK protection, rather than pan-HLA deletion or global T-cell suppression. Table 3 summarizes the corresponding evidence and product-specific constraints.

## 5. NK-Cell Surveillance

Natural killer (NK)-cell surveillance becomes particularly important when antigen presentation is reduced to limit recipient T-cell recognition. HLA class I molecules are alloantigens for recipient CD8^+^ T cells but also provide inhibitory input to NK cells through killer-cell immunoglobulin-like receptors (KIRs) and CD94/NKG2A. HLA reduction can therefore exchange one immune pressure for another rather than uniformly decrease immunogenicity [25,32].

Poxviruses reveal that this problem extends beyond missing-self recognition. Orthopoxvirus MHC class I-like protein (OMCP) directly antagonizes NKG2D, whereas poxviral hemagglutinin (HA) differentially modulates NKp30 and NKp46 [58,59]. These mechanisms identify activating-receptor interfaces that remain relevant even when inhibitory HLA signaling is preserved. The corresponding schematic therefore focuses on NKG2D and natural-cytotoxicity-receptor recognition and on the corresponding human intervention levels. The inhibitory HLA background remains a separate constraint on how these activating interfaces are interpreted.

The evidence also spans different experimental depths. Viral infection, binding, and structural studies establish what OMCP and HA do; perturbation studies in human cells show which ligand states can be manipulated; and only experiments in CAR cells or differentiated graft-relevant products establish compatibility with a therapeutic product. The comparison below keeps these evidence levels separate.

### 5.1. Poxviral Control Points Across NK-Cell Recognition

The first control layer is the balance between reduced HLA-dependent T-cell recognition and retained inhibitory NK signaling. HLA-E expression can restore CD94/NKG2A-dependent inhibition after B2M disruption, whereas selective HLA-A/HLA-B deletion with HLA-C preservation retains part of the KIR-ligand architecture [36,37]. Antigen-processing interventions can disturb this balance even without complete HLA deletion. As discussed in Section 4, MC080 reduces classical HLA-I together with HLA-E while suppressing CD8^+^ T-cell activation, showing that the relevant endpoint of TAP-, tapasin-, or trafficking-level intervention is the residual inhibitory HLA state rather than HLA-I reduction alone [150,155].

OMCP defines a second and mechanistically independent layer. It is a secreted MHC-I-like protein that binds NKG2D with high affinity, competes with cellular ligands for receptor binding, and inhibits NKG2D-dependent NK-cell cytotoxicity [58,175]. The important principle is that induced-self recognition through MICA, MICB, and ULBP/RAET1-family ligands can remain active even when the HLA/KIR/NKG2A balance has been corrected. NKG2D-ligand state is also dynamic. Cas9-induced DNA damage activates p53 in hPSCs, whereas separate p53 and senescence studies show increased ULBP1/ULBP2 or MICA/ULBP2 expression; differentiation, hypoxia, and inflammatory cytokines can further change the dominant activating-ligand state in human cells [176,177,178,179,180,181]. These observations justify testing an editing- or stress-associated NKG2D phenotype; they do not imply that every edited product acquires one.

Poxviral HA defines a third layer at the natural cytotoxicity receptor interface. HA binds NKp30 and NKp46 and has receptor-specific functional effects: in the experimental systems examined, HA reduced NKp30-mediated activation while enhancing NKp46-mediated activation, demonstrating that the two receptors cannot be treated as one generic activating pathway [59]. Human ligands reinforce this distinction. Membrane B7-H6 activates NKp30, BAG6 activity depends on whether the ligand is extracellular-vesicle-associated or soluble, and ER stress can expose ecto-calreticulin (ecto-CRT) for NKp46 recognition [182,183,184,185,186]. The poxviral lesson is therefore receptor- and ligand-form specificity rather than broad NCR inhibition.

Finally, receptor engagement must be converted into productive cytotoxic contact. Adhesion molecules and antibody-dependent CD16 signaling therefore constitute a downstream execution layer. This is not a separate poxviral NK-receptor mechanism: CPXV14 provides an FcγR-linked bridge from Section 4, whereas human CD54/CD58 and CD64 studies directly show that immune contact and antibody-dependent injury can be modified at the therapeutic-cell interface [44,73,160].

### 5.2. Human Interventions Show That NK Interfaces Are Directional and Product Dependent

Human engineering studies support preservation of selected inhibitory signals rather than complete removal of NK-recognition capacity. In gene-edited allogeneic CAR-T cells, an HLA-E single-chain trimer presenting the VMAPRTLIL signal peptide protected against NKG2A-positive NK populations while producing negligible activation of NKG2C-positive populations; additional CD54/CD58 deletion further increased resistance across heterogeneous NKG2A/NKG2C populations [187]. LLT1/CD161 signaling provides a separate non-HLA inhibitory comparator in allogeneic CAR cells [69]. These approaches differ mechanistically from OMCP or HA because they increase inhibitory input rather than suppress an activating NK receptor.

Human perturbation of the NKG2D-ligand axis shows why ligand abundance, surface retention, and extracellular availability must be considered separately. CREBBP/CBP and EP300/p300 loss in human HEK-293 cells reduced basal and stress-induced MICA/B and ULBP2 expression and reduced the associated increase in primary-NK-cell killing [188]. By contrast, decitabine-induced TIMP3 reduced ADAM17-dependent shedding of MICA, MICB, and ULBP2 in human AML cells and thereby increased surface-mediated NKG2D recognition and NK cytotoxicity [189]. Thus, reducing soluble-ligand release can increase rather than decrease NK pressure. Extracellular ULBP biology is also directly manipulable: an Fc-enhanced antibody against ULBP6/2/5 neutralizes soluble ULBP6 while adding FcγRIIIa-dependent effector activity [190]. These studies establish intervention depth, but they are primarily cancer-model evidence rather than validated hypoimmune-product strategies.

Multiplex hPSC engineering provides closer graft-proximal evidence. MICA and MICB were disrupted together with B2M, TAP1, CIITA, and CD74 in a multilayer immune-evasion platform [191]. Persistent growth in immunocompetent mice still required additional NK-inhibitory and complement-control modules. Because the study used multiplex editing, xenogeneic transplantation, and teratoma formation, it establishes the tractability of MICA/MICB manipulation but does not isolate their contribution to protection of a differentiated human allograft.

The NKp30 axis has stronger CAR-relevant evidence. Activated human T cells, including patient-infused CD19 CAR-T cells, can express B7-H6 and become targets of NKp30-mediated surveillance. Genetic NCR3LG1/B7-H6 deletion reduced NKp30-dependent cytolysis, increased T-cell proliferation, and improved CAR-T persistence and antitumor activity in humanized mice [192]. This makes target-cell ligand modification a closer translational analogue of HA-mediated NKp30 interference than receptor-wide NKp30 blockade.

NKp46 provides an important opposite constraint. Senescent human A549 cells increased surface ecto-CRT, and antibody blockade of ecto-CRT reduced NK-cell killing; disruption of NCR1/NKp46 on responding NK cells produced the same directional effect [186]. Ecto-CRT is therefore a functional stress-surveillance ligand, but reducing this interface could also weaken recognition of stressed or abnormal cells. Intracellular CALR biology remains distinct and is considered with the peptide-loading complex in Section 4.

Contact-level interventions add another product-specific trade-off. CD54/CD58 deletion can reduce rejection of engineered CAR cells, but ICAM1/LFA-1 and CD58/CD2 also contribute to productive CAR–target adhesion and immune-synapse formation [73,193]. Similarly, CD64 can reduce IgG-dependent NK and complement-mediated injury without preventing antibody formation itself [44]. Protection at the execution layer therefore cannot be interpreted independently of therapeutic-cell function.

### 5.3. Product-Specific Interpretation of NK-Cell Surveillance

For allogeneic CAR products, the most compatible NK-control strategies are those restricted to the therapeutic cell and its dominant recognition interface. Preserving selected inhibitory HLA signals, modifying an induced target-cell ligand such as B7-H6, or attenuating excessive host-cell contact is more compatible with CAR biology than constitutive blockade of NKG2D, NKp30, or NKp46. The latter receptors participate in antiviral and antitumor surveillance outside the CAR-cell compartment. Contact-level engineering must also preserve CAR–target conjugation, repeated killing, expansion, trafficking, and clinical eliminability [44,73,187,192].

For iPSC-derived regenerative grafts, the activating-ligand state can change during differentiation and after implantation. Neural differentiation can add B7-H6/NKp30 recognition to an initially NKG2D-dominated phenotype, hypoxia can increase selected NKG2D ligands, and ER stress can expose ecto-CRT for NKp46-dependent surveillance [179,180,186]. Multiplex MICA/MICB editing demonstrates that this axis is engineerable, but current evidence does not establish a universal stress-ligand configuration for differentiated regenerative grafts [191].

Within the review’s translational hierarchy, preservation or restoration of selected inhibitory HLA signals and validated contact-level host interventions falls within Tier 1–2. Ligand-level NKG2D tuning and HA-inspired NKp30/NKp46-interface modulation are Tier 2 because the mechanistic and human-cell evidence is strong but final-product validation remains incomplete. Constitutive receptor-wide blockade of NKG2D, NKp30, or NKp46 is Tier 3 because it extends beyond graft protection into broader NK surveillance. The design rule is therefore to identify and control the dominant residual NK-recognition interface in the final therapeutic product rather than suppress NK-cell recognition globally. These evidence levels and constraints are summarized in Table 4.

**Figure 5:**
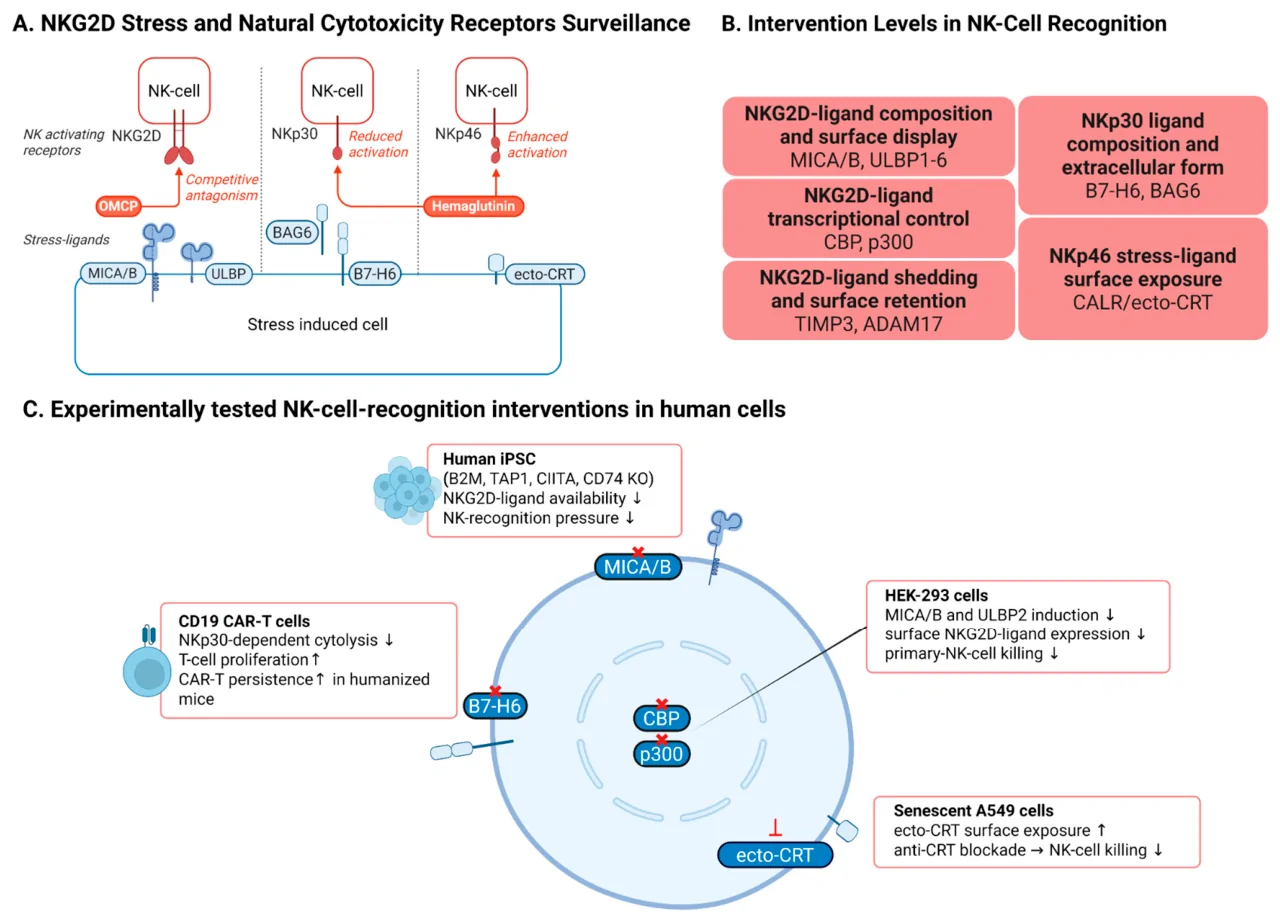
Activating NK-cell receptor interactions and the corresponding evidence in human cells. (A) Poxviral protein–receptor interactions and their reported functional effects: OMCP competitively antagonizes NKG2D and inhibits NKG2D-dependent cytotoxicity; poxviral hemagglutinin binds NKp30 and NKp46, reducing NKp30-mediated activation while enhancing NKp46-mediated activation in the experimental systems examined [58,59]. Host ligands are shown to identify the respective receptor interfaces. (**B**) Corresponding host-pathway levels encompass ligand composition and display, transcription, shedding, extracellular form, and stress-associated surface exposure. (**C**) Findings from the indicated human-cell models, including pluripotent cells, CAR-T cells, HEK-293 cells, and senescent A549 cells. These systems provide different levels of therapeutic relevance; persistence in humanized mice is a preclinical outcome, and mechanistic findings in other cell lines do not establish efficacy in a final therapeutic product [186,188,191,192]. The schematic concerns activating-receptor interfaces and should be interpreted alongside the separate inhibitory signals associated with HLA-I. Red arrows in panel A indicate viral protein–receptor interactions; the adjacent labels specify the functional effects and the arrowheads do not uniformly denote activation. Red T-bars indicate inhibition or blockade, and red crosses indicate gene disruption. Black leader lines link interventions to their study callouts; upward and downward arrows in callouts indicate increased and decreased expression or reported outcomes, respectively. Host components are blue and poxviral proteins red/orange. BAG6, BCL2-associated athanogene 6; ecto-CRT, cell-surface calreticulin; NK, natural killer; OMCP, orthopoxvirus MHC class I-like protein.

Figure 5 illustrates the corresponding receptor interfaces and human-cell findings.

## 6. Cytokine, Chemokine, Complement, Inflammatory Signaling, and Death Pathways

Control of IFN-dependent remodeling, antigen presentation, T-cell recognition, and NK-cell surveillance does not by itself protect an allogeneic product from inflammatory injury. Cytokines can alter therapeutic-cell function or trigger death; chemokines determine where recipient leukocytes accumulate; antibodies and complement injure the cell surface; and blood contact couples complement to coagulation, platelets, neutrophils, and neutrophil extracellular traps (NETs) [7,8,16,17,194,195]. These pressures form an inflammatory-survival layer that remains relevant after the recognition barriers discussed in Section 3, Section 4 and Section 5 have been reduced.

**Figure 6:**
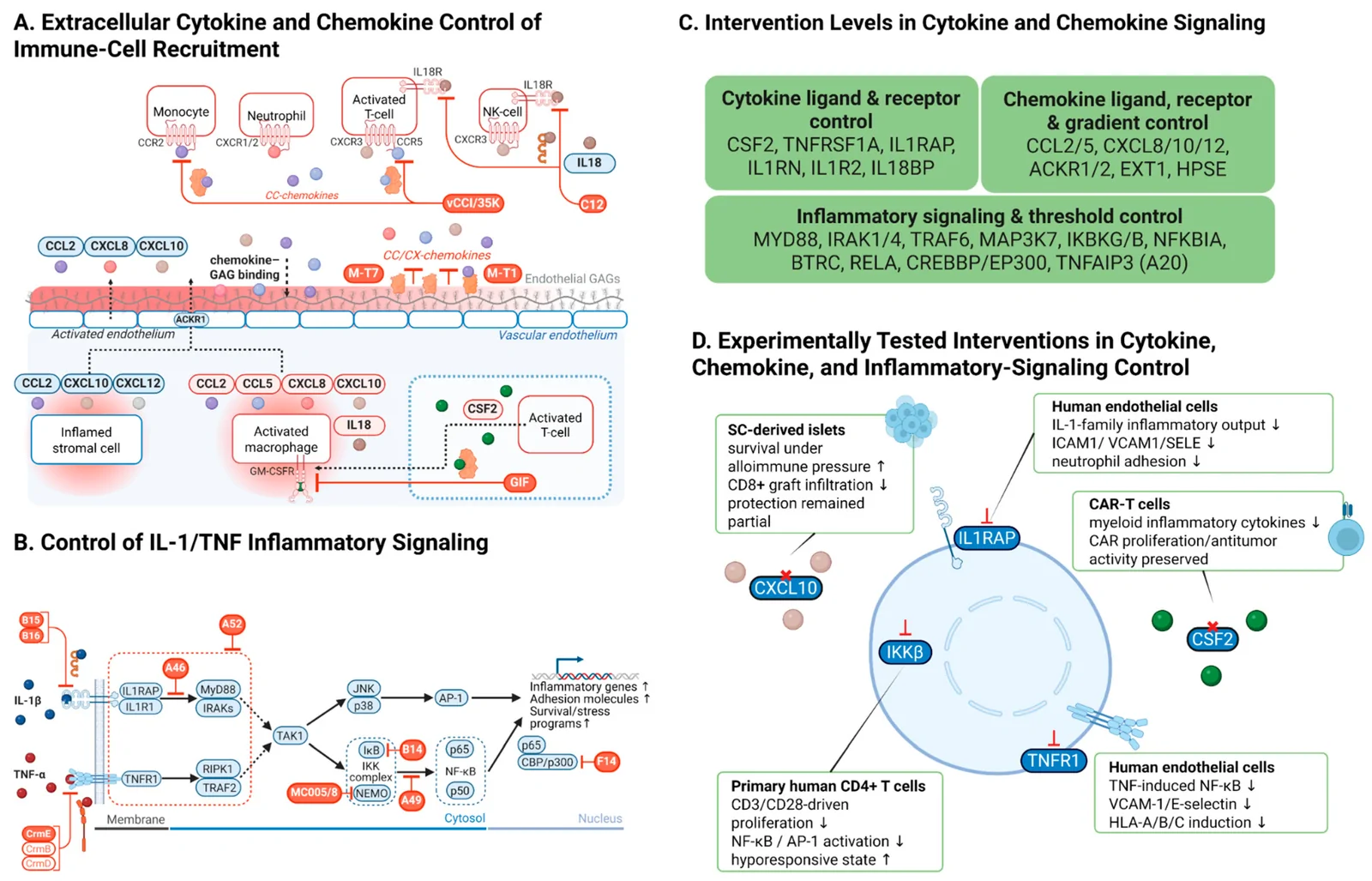
Poxviral modulation of inflammatory mediators and corresponding human-cell findings. (**A**) Extracellular cytokine and chemokine interactions, including effects on chemokine positioning at endothelial glycosaminoglycans (GAGs). The GIF example represents a species-restricted cytokine-binding mechanism and is not evidence of equivalent activity against human GM-CSF. (**B**) IL-1 and TNF signaling converge on pathways controlling inflammatory transcription, with representative poxviral effects shown at several levels. (**C**) Corresponding host-pathway domains encompass cytokine responses, chemokine gradients, and inflammatory signaling. (**D**) Published findings in the indicated CAR-T, endothelial, islet, and primary T-cell systems. The callouts include both favorable outcomes and limitations: islet protection remained partial, and reduced inflammatory signaling could coincide with diminished T-cell proliferation. Each finding belongs to its stated model and does not establish a uniform effect across therapeutic lineages. Red T-bars denote inhibition or blockade; red crosses, gene disruption; solid black arrows, pathway progression; dashed or dotted connectors, indirect or schematic links between the indicated processes; upward and downward arrows, increased and decreased expression or reported outcomes, respectively. Black leader lines link interventions to their study callouts. Host components are blue/grey, poxviral proteins red/orange, and host-pathway domains green. GM-CSF, granulocyte–macrophage colony-stimulating factor; IL, interleukin; SC, stem-cell-derived; TNF, tumor necrosis factor.

**Figure 7:**
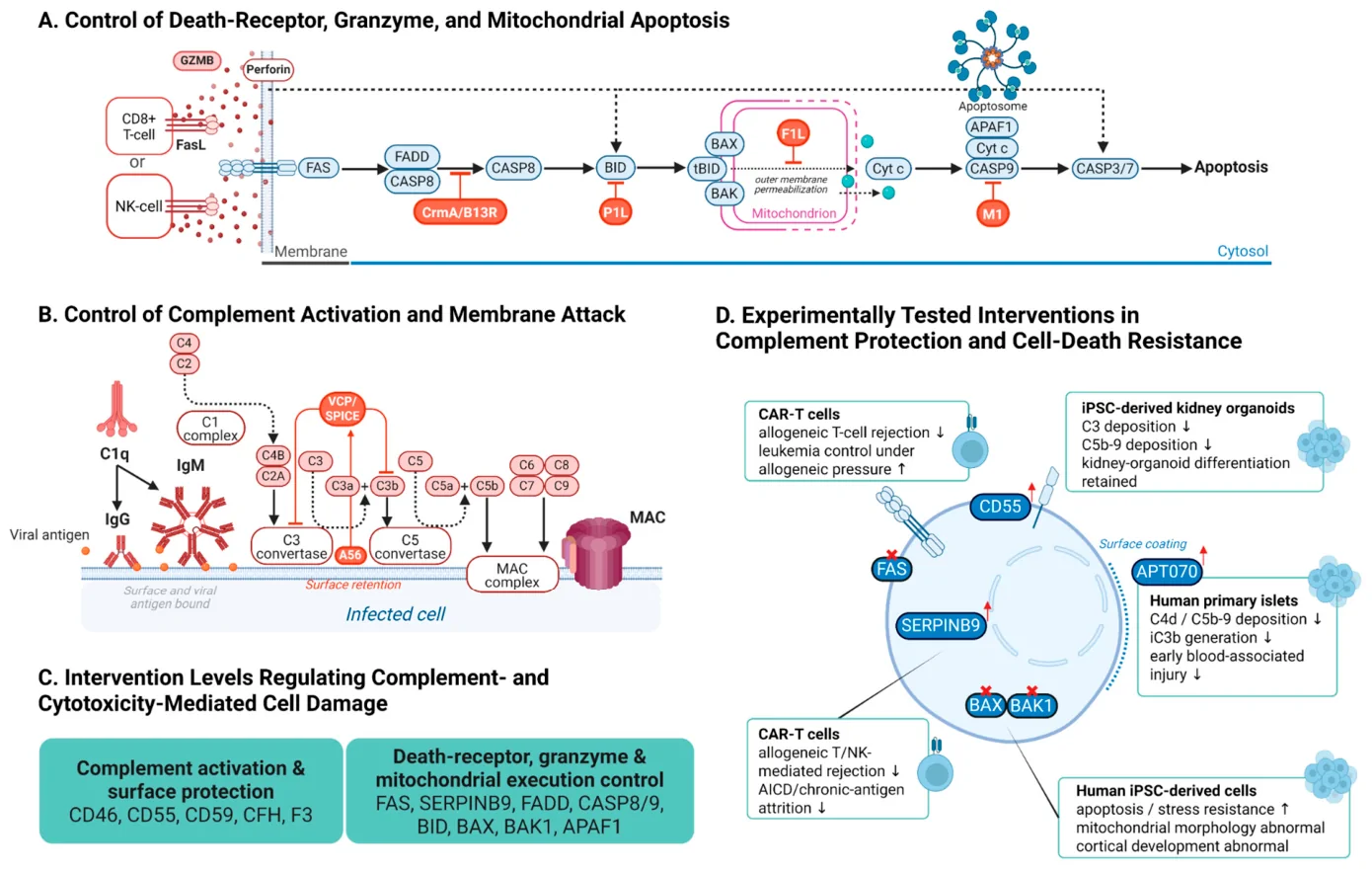
Complement-associated injury and cell-death pathways in relation to poxviral mechanisms and human-cell evidence. (**A**) Death-receptor, granzyme-associated, mitochondrial, and apoptosome-linked pathways, with representative poxviral inhibitory effects. (**B**) Complement amplification and membrane attack, together with poxviral complement regulation and retention of a regulator near the infected-cell surface. (**C**) Host-pathway domains distinguish surface complement protection from resistance at different stages of cell-death execution. (**D**) Published findings in the indicated CAR-T cells, iPSC-derived kidney organoids, human primary islets, and iPSC-derived cells. Complement-associated deposition and injury are distinguished from resistance to cytotoxic or stress-induced cell death. The developmental and mitochondrial abnormalities shown in the lower-right callout underscore that increased survival does not establish preserved cell function or clinical safety. The panel combines evidence from separate models and does not show a validated combined product. Red T-bars denote inhibition or blockade; red crosses, gene disruption; solid black arrows, pathway progression; dashed or dotted black arrows, schematic links between the indicated processes; upward and downward arrows, increased and decreased expression or reported outcomes, respectively. Black leader lines link interventions to their study callouts. Host-pathway domains are teal. AICD, activation-induced cell death; Cyt c, cytochrome c; GZMB, granzyme B; MAC, membrane attack complex.

Poxviruses expose several mechanistically distinct ways to control this layer (Figure 6 and Figure 7). Secreted proteins intercept cytokines before receptor engagement, chemokine-binding proteins remove ligands or disrupt glycosaminoglycan (GAG)-dependent positioning, complement regulators act close to the protected surface, intracellular proteins alter inflammatory-signaling thresholds, and viral serpins or Bcl-2-like proteins delay terminal cell death [50,51,52]. Figure 6 focuses on extracellular cytokine and chemokine control together with IL-1/TNF-driven inflammatory signaling, whereas Figure 7 separates complement-mediated surface injury from receptor-, granzyme-, mitochondrial-, and apoptosome-level death execution. These levels are not interchangeable: changing a distributed ligand field, donor-cell responsiveness, the graft surface, or terminal death execution affects different compartments and carries different functional costs.

The preferred direction also differs between products. CAR immune cells require chemokine-guided trafficking, cytokine responsiveness, NF-κB-linked activation, proliferation, and cytotoxicity. Stationary iPSC-derived regenerative grafts may benefit more from local inflammatory buffering or short peri-engraftment protection, but still require vascularization, tissue-specific function, immune surveillance, and eliminability of abnormal cells. The relevant goal is therefore selective, compartment-defined protection rather than general inflammatory suppression.

### 6.1. Poxviral Control Points Across the Inflammatory-Survival Layer

Extracellular cytokine interception is the most direct poxviral strategy in this section. Crm proteins capture TNF before it reaches cellular TNFRs, whereas B15/B16-like proteins and C12 intercept IL-1β and IL-18, respectively [196,197,198,199]. CrmB and CrmD are broader because their C-terminal SECRET domains also bind selected chemokines [200,201]. ORFV GIF extends the same principle to GM-CSF, but its weak neutralization of human GM-CSF makes it a species-restricted precedent rather than a human therapeutic module [202,203].

Chemokine availability and chemokine positioning are separate control problems. vCCI/35K and M-T1 mainly prevent productive chemokine-receptor engagement, whereas M-T7, A41, and E163 interfere with chemokine interaction with GAGs and therefore with immobilized gradients on endothelium or extracellular matrix [204,205,206,207,208,209]. This distinction is functional, not semantic: chemokines can retain receptor activity in solution yet lose effective leukocyte recruitment when GAG-dependent presentation is disrupted [210].

Poxviruses also suppress inflammatory signaling at multiple intracellular depths. A46 and A52 act at proximal TLR/IL-1R signaling complexes; MC005 and MC008 converge on NEMO-dependent IKK activation; B14 inhibits IKKβ; A49 interferes with β-TrCP-dependent IκB turnover; and F14 acts distally at the RelA/p65–CBP/p300 coactivation interface [211,212,213,214,215,216]. These proteins show that inflammatory output can be altered without using one generic “NF-κB blockade.” They also define a safety boundary: A52 additionally disrupts SNAP29-dependent autophagosome–lysosome fusion, demonstrating that a potent viral signaling inhibitor may couple inflammatory suppression to unrelated homeostatic functions [217].

Complement control is spatially different again. VCP/VICE and SPICE regulate C3/C4-dependent amplification, while A56 retains VCP near the infected-cell surface [218,219,220]. The transferable principle is therefore cell-proximal complement regulation rather than systemic complement suppression, especially for blood-exposed products in which complement interacts with antibody binding, coagulation, platelets, and neutrophils.

At the deepest level, poxviruses delay death execution after inflammatory or cytotoxic attack has already occurred. B13R and CrmA interfere with inflammatory or extrinsic caspase pathways; F1L acts before mitochondrial commitment; MPXV P1L binds the BH3-only protein BID at the death-receptor-to-mitochondrial interface; and M1 limits apoptosome-associated caspase-9 and downstream caspase-3 processing [221,222,223,224,225,226]. N1 is not used as a core survival mechanism because its early anti-apoptotic assignment was not reproduced independently [227,228]. The deeper the intervention lies in death execution, the more strongly survival benefit must be separated from preservation of damaged or otherwise unsafe cells.

### 6.2. Human Perturbation Studies Define Compartment and Direction

TNF and IL-1 studies show why ligand interception, receptor attenuation, and downstream threshold control cannot be treated as substitutes. Extracellular TNF blockade improved engraftment in allogeneic hepatocyte transplantation, and engineered human MSCs could produce an active sTNFRII–Fc sink [229,230]. TNFR1 attenuation in human endothelial cells reduced TNF-induced HLA-I and adhesion molecules, whereas complete TNFR1 perturbation produced compensatory effects and TNFR2 loss impaired MSC immunoregulatory and angiogenic functions [231,232,233]. High etanercept exposure also impaired human-islet function [234]. For IL-1, IL1RN and IL1R2 demonstrate extracellular or decoy-style control, while IL1RAP blockade in human endothelial cells reduced IL-1-family inflammatory signaling, adhesion-molecule expression, permeability, and neutrophil adhesion [235,236,237]. IL1RAP is shared by several IL-1-family receptor complexes, so this remains mechanistic human-endothelial evidence rather than validated final-product engineering.

IL-18 and GM-CSF illustrate stronger product divergence. IL18BP is a valid reversible extracellular neutralization strategy, yet IL-18 can enhance CAR-cell activity and endogenous IL18BP can restrict that response [238,239,240]. GM-CSF provides direct CAR evidence in the opposite direction: CSF2 deletion or extracellular GM-CSF neutralization reduced monocyte inflammatory output while preserving antitumor activity in the tested systems, whereas loss of GM-CSF-receptor signaling impaired the intended function of iPSC-derived macrophages [241,242,243,244]. The same cytokine axis can therefore favor source control in one product and require receptor preservation in another.

Chemokine studies make spatial access a first-order variable. M-T7 reduced leukocyte infiltration and chronic graft injury in transplantation models, but survival benefit varied with model and delivery format; short donor-graft pretreatment also reduced subsequent inflammation [53,245,246]. Membrane-tethered 35K inhibited recruitment in one setting but failed to do so in another [247,248]. By contrast, CXCL10 depletion in human stem-cell-derived islets provides direct differentiated-product evidence that selective source control can reduce immune attack, although protection remained incomplete [88]. These studies favor spatially validated or ligand-selective control over long-lived broad chemokine sinks and show why soluble binding assays alone cannot establish effective interception in tissue [210,249,250].

Complement and blood-interface studies provide the strongest case for local surface or pericellular protection. Recombinant VCP has autonomous activity outside productive infection and reduced complement-associated injury in graft-relevant systems [251,252,253]. Human comparators are more product-proximal: CD55 overexpression reduced antibody-triggered complement deposition in iPSCs and kidney organoids; CFH overexpression in human iPSC-derived choroidal endothelial cells increased local factor-H production and reduced membrane-attack-complex deposition; CD64 reduced IgG-dependent killing; and temporary APT070 coating protected human islets at the blood interface [44,83,84,254]. Coagulation and platelet studies nevertheless show that less blood interaction is not automatically better: interventions act on different stages of IBMIR-like injury, and GPVI-dependent platelet stabilization can support islet engraftment even while excessive thromboinflammation is damaging [86,255,256,257].

Human perturbation studies also narrow the interpretation of intracellular poxviral mechanisms. A20-mediated feedback can reduce inflammatory injury without erasing NF-κB signaling, whereas central NF-κB activity supports CAR activation and persistence [89,258]. Inflammasome evidence further shows that the dominant compartment may lie outside the therapeutic cell: CAR-associated pyroptotic amplification can arise from target-cell GSDME and recipient macrophages, while cardiac-allograft studies identified GSDMD- and AIM2-associated programs predominantly in recipient inflammatory macrophages [259,260,261]. Permanent product-cell inflammasome editing would therefore miss the causal compartment in such settings.

Death-execution studies show the clearest rise in control burden with intervention depth. FAS perturbation and engineered SERPINB9 directly improved resistance of allogeneic CAR cells to selected T- or NK-mediated killing, and SERPINB9 protection remained compatible with an inducible suicide switch [48,74,262]. CrmA expression provides a poxviral precedent for autonomous graft-level protection, but its caspase inhibition is broader [221,263,264]. Human pluripotent-cell evidence sharpens the boundary: BAX/BAK1 double knockout increased survival after mitochondrial stress but also produced abnormal mitochondrial morphology and aberrant cortical structures after neural differentiation [265]. Human-islet studies similarly show that lower apoptotic readouts do not guarantee a better product: XIAP was cytoprotective, whereas caspase-3 inhibition or sustained BCL2/BCL-xL activity could impair endocrine processing, metabolism, or function [266,267,268]. Survival must therefore be interpreted together with lineage-specific potency and eliminability.

### 6.3. Product-Specific Synthesis and Tier Logic

For allogeneic CAR products, the most compatible interventions are selective and cell- or source-restricted. GM-CSF source control and selective FAS or granzyme-pathway resistance already have direct CAR evidence. CD64 or complement protection may be useful when antibody-dependent clearance is a demonstrated pressure. By contrast, broad chemokine sequestration can compromise tumor homing, endothelial crossing, and matrix entry, while central NF-κB inhibition can impair activation and persistence [44,74,241,258,269,270]. A CAR-directed inflammatory module should therefore target a demonstrated harmful signal without disabling trafficking, repeated killing, cytokine responsiveness, or rapid clinical depletion.

For iPSC-derived regenerative grafts, the strongest logic is local and often time-limited. TNF/IL-1 buffering, ligand-selective chemokine control, and surface or pericellular complement regulation can protect the early graft environment without requiring permanent intracellular blindness. Blood-interface modules are most relevant to intraportal, intravascular, endothelial, or islet-like products and should not be transferred automatically to locally implanted tissues. Receptor or death-pathway changes must also be tested after differentiation because TNFR2, GM-CSF receptors, NF-κB, and BCL2-family proteins can contribute directly to vascular support, lineage maturation, metabolism, or tissue function [233,244,254,268,271].

The Tier hierarchy follows intervention depth, evidence proximity, and controllability. Direct host-derived strategies such as CAR-cell CSF2 source control, CD55/CD64 surface protection, or selective FAS/SERPINB9 resistance are closest to Tier 1–2. Local poxvirus-inspired cytokine or chemokine interception, VCP/SPICE-like complement control, A20-like inflammatory tuning, and compartment-defined inflammasome modulation remain predominantly Tier 2 because final-product validation is incomplete. Constitutive broad cytokine or chemokine sinks, central NF-κB or inflammasome ablation, CrmA-like broad caspase inhibition, and permanent mitochondrial or apoptosome resistance remain Tier 3. Figure 6 and Figure 7 therefore separate two complementary design problems: control a demonstrated extracellular source, receptor, or inflammatory threshold before suppressing a central signaling hub; localize complement regulation to the exposed surface; and use selective FAS- or granzyme-pathway protection before considering mitochondrial or apoptosome-level resistance. Table 5 summarizes these mechanisms and their evidence boundaries.

## 7. From Poxviral Mechanisms to Conditional Defense-in-Depth

Section 3, Section 4, Section 5 and Section 6 reveal a common feature of poxviral immune evasion: the same immune pressure can often be controlled at several different levels. IFN can be intercepted outside the cell or attenuated at receptor, STAT, or sensing levels. Antigen presentation can be changed through peptide supply, peptide loading, or HLA trafficking. NK recognition depends not only on missing-self but also on activating receptor–ligand interactions. Cytokines, chemokines, complement, inflammatory signaling, and terminal cell death provide further routes of injury after T- and NK-cell recognition has been reduced.

On the basis of these findings, we propose a conditional defense-in-depth framework. It is not a fixed multilayer genotype. Each added module should address a residual barrier demonstrated in the final therapeutic product. Mechanism-level Tier 1–3 assignments remain in Table 2, Table 3, Table 4 and Table 5; here, mechanisms from all tiers are used when they reveal a distinct and useful level of control.

Four principles guide the framework. Antigen-presentation changes must be interpreted together with NK-cell recognition. IFN modulation should match the compartment that drives the unwanted response. Extracellular inflammatory protection should preserve spatial control rather than create unrestricted cytokine or chemokine suppression. Finally, protection from terminal killing should be added only when upstream control is insufficient and must remain compatible with independent elimination.

### 7.1. Direct Viral-Protein Expression: What Should Be Borrowed?

Using an intact poxviral protein is one possible implementation, but it is not the only way to translate a viral mechanism. The poxviral literature already includes several forms of autonomous activity outside productive infection. B18/B19R remains active as recombinant protein or when supplied through mRNA and can create a locally concentrated type I IFN sink through glycosaminoglycan-dependent surface binding [111,124,125,127]. Myxoma-virus M-T7 and Serp-1 have also reduced inflammatory or vascular injury when delivered as recombinant proteins in transplantation models, and brief M-T7 pretreatment of donor tissue reduced subsequent graft inflammation and scarring [53,54,55,246]. These studies support autonomous poxviral activity, but not constitutive expression in a final CAR or iPSC-derived product.

The main translational question is therefore what part of the viral solution should be preserved. Sometimes the whole protein may be useful. In other cases, the binding interface, spatial behavior, or host-control principle may be more valuable than the viral molecule itself. This distinction is already visible experimentally. The STAT1-binding β-hairpin of VACV 018 has inspired synthetic macrocyclic peptides that retain STAT1 binding and compete with receptor docking [277]. These peptides are pharmacological probes rather than cell-therapy interventions, but they provide a direct example of structure-guided biomimicry of a poxviral mechanism.

A second example comes from CrmB and CrmD. Their natural architecture combines TNF capture with chemokine binding through a separate SECRET domain. This principle was transferred to an etanercept-based fusion protein, producing a bifunctional TNF/chemokine inhibitor [278]. Here again, the useful feature was the modular architecture rather than the intact viral receptor.

Other mechanisms remain largely underused in low-immunogenic cell products. CPXV203 acts after MHC-I assembly by retrieving mature complexes from the secretory pathway rather than eliminating HLA synthesis [151,172]. Intracellular retention itself is an engineerable concept: ER-retained protein-expression blockers have been used in primary human NK cells to remove NKG2A from the cell surface without preventing its synthesis [279]. This is not direct CPXV203 biomimicry, but it shows that selective control of surface-protein trafficking is technically possible. Applying that principle to HLA composition could provide a different route from permanent HLA deletion.

Direct viral-protein expression is therefore most convincing when the protein acts autonomously against the human target, its molecular action is well defined, its expression preserves the required function of the final product, and its activity can be confined to an appropriate place and time. The viral protein itself must also be evaluated as a new antigen, including pre-existing recognition and treatment-emergent antibody or T-cell responses [280,281]. Persistent immunity after vaccinia vaccination provides context for pre-existing antiviral recognition, but does not establish recognition of each candidate immunomodulatory protein [282]. When these conditions are not met, sequence minimization, deimmunization, structure-guided redesign, a host-derived functional analogue, or transient and localized delivery may reproduce the useful function with less added complexity [283].

### 7.2. Conditional Defense-in-Depth for Allogeneic CAR Products

Allogeneic CAR-T and CAR-NK cells are mobile immune effectors. Immune protection is useful only if the product retains trafficking, CAR–target-cell conjugation, repeated killing, antigen-driven expansion, required cytokine signaling, and rapid clinical depletion.

The foundation is therefore a functional and controllable product. For CAR-T cells, donor-TCR control addresses GVHD but does not protect the product from recipient immunity. Editing completeness, genome integrity, potency, and an independent OFF mechanism remain separate requirements [47,284].

If HLA-I or peptide presentation is reduced, T-cell and NK-cell control become one problem. Section 4 and Section 5 show why. CPXV012, MC080, and CPXV203 act at peptide transport, peptide loading, and post-assembly HLA trafficking, respectively [149,150,151]. These mechanisms suggest that antigen presentation can be tuned at different depths instead of being treated as an HLA on/off switch. CPXV203 is particularly interesting because post-assembly trafficking could, in principle, allow selective control of surface HLA composition. The PEBL study provides an independent technical precedent for engineering intracellular retention of a chosen surface protein in primary human immune cells [279].

The NK response must then be measured in the phenotype created by that intervention. MC080 can reduce HLA-E together with classical HLA-I, directly linking peptide-loading control to NK susceptibility [155]. OMCP and poxviral hemagglutinin add another lesson: after missing-self has been addressed, NKG2D and NKp30/NKp46 can remain independent routes of recognition [58,59]. Thus, the aim is not simply to restore one inhibitory ligand, but to determine which inhibitory and activating NK signals dominate in the final manufactured CAR product.

If recipient immunity still clears the cells, protection can move downstream. Macrophage, antibody, and productive-contact mechanisms should be treated separately because they act through different interfaces. CD47, CD64, and CD54/CD58 studies provide direct product-level examples [33,44,73]. CPXV14 adds a poxviral connection between immune complexes, FcγR-positive accessory cells, and adaptive amplification [160]. The practical question is which of these routes actually limits the engineered CAR cell, not how many can be blocked simultaneously.

IFN and inflammatory control should follow the same compartment logic. B18/B19R changes the extracellular type I IFN field; 018, VH1, and C6 act at different steps inside the responding cell; poxin, E5, and MC089 act further upstream on innate sensing or IFN induction [113,114,115,116,124,130,285]. The 018-derived peptide work illustrates how one such intracellular mechanism can be reduced to a defined binding motif rather than copied as a full viral protein [277]. Which depth is useful depends on whether the limiting problem is extracellular cytokine exposure, CAR-cell responsiveness, or cell-intrinsic inflammatory signaling. This distinction matters because strong type I IFN exposure can promote CAR-cell attrition, whereas IFN-γ signaling in tumor targets can support CAR–target adhesion and killing [129,141].

Terminal killing forms a final optional layer. FAS disruption and engineered SERPINB9 show directly that receptor- and granule-associated killing can be attenuated in allogeneic CAR cells [48,74]. Poxviral B13R, F1L, and M1 show that protection can also be placed at inflammatory/extrinsic caspases, mitochondrial commitment, or apoptosome output [222,224,226]. These are different intervention depths, not interchangeable anti-apoptotic strategies.

The CAR architecture is therefore conditional. HLA or peptide-presentation attenuation creates a paired NK requirement. Macrophage, antibody, inflammatory, or terminal-killing modules are added only when those mechanisms remain limiting in the final CAR product.

### 7.3. Conditional Defense-in-Depth for iPSC-Derived Regenerative Grafts

For iPSC-derived regenerative grafts, the starting point is a stable final lineage with preserved tissue-specific function, acceptable genome integrity, and an independent way to eliminate abnormal cells [8,286]. The protective architecture should then follow the actual graft site and the immune pressures measured in that differentiated product.

As in CAR products, attenuation of HLA-I or peptide presentation creates a paired T/NK problem. CPXV012, MC080, and CPXV203 again show that antigen presentation can be modified at different processing and trafficking levels [149,150,151]. The relevant unit is the final differentiated and stressed graft because differentiation and inflammation can change both inhibitory HLA and activating NK ligands. A35R, M2, and B22 extend the adaptive design space beyond MHC-I by pointing to MHC-II presentation, costimulation, and responder-side T-cell activation as separate levels of control [158,161,173]. B22 is especially interesting as a discovery mechanism: identifying the host receptor or molecular target responsible for this effect may reveal a T-cell control point not exposed by standard HLA engineering.

The next barrier may be the graft interface. For blood-contacting products, poxviral complement regulators show the value of concentrating protection at the cell surface. SPICE inhibits human complement, while A56 retains VCP at infected-cell membranes [219,220]. A similar spatial principle has been reproduced with host molecules: factor H painted onto MSC surfaces reduced local complement activation and complement-dependent neutrophil injury [287]. This is an example of convergent biomimicry—the viral solution identifies the useful geometry, while a host-derived molecule provides the implementation.

M-T7 and Serp-1 illustrate a second interface strategy. Rather than changing the graft permanently, short-course or graft-proximal treatment can alter the inflammatory environment during the period when tissue injury is greatest [55,246]. This approach is especially relevant to long-lived regenerative products, where a mechanism needed during implantation may not need to remain active for the lifetime of the graft.

Inflammatory protection can then be divided into signal strength and spatial information. B18/B19R can create a local type I IFN sink, whereas 018, VH1, and C6 reduce signaling inside the graft cell [113,114,124,130]. Poxin, E5, and MC089 identify another option: reduce graft-derived innate alarm upstream of IFN production [115,116,285]. These choices should not be collapsed into one “IFN-resistant” phenotype. The appropriate depth depends on whether the residual problem is recipient-derived cytokine, graft-cell responsiveness, or graft-derived sensing. Human endocrine, retinal, and cardiac systems already show that IFN-induced immune remodeling differs between final lineages [87,103,104,105].

Chemokine control provides an even more spatial poxviral lesson. vCCI/35K and M-T1 mainly reduce chemokine availability, whereas M-T7, A41, and E163 interfere with GAG-dependent chemokine presentation [204,205,206,207,209]. The distinction matters because leukocyte recruitment depends not only on how much chemokine is produced, but also on where it is immobilized. This suggests an underused engineering goal: modify the spatial presentation of selected rejection-driving chemokines rather than eliminating the whole chemokine system. Global loss of the GAG platform would not be equivalent because heparan sulfate also supports stem-cell signaling, differentiation, and tissue organization [288].

CrmB and CrmD show that two extracellular functions can also be combined. Their TNF-binding and SECRET chemokine-binding domains inspired a bifunctional etanercept-based inhibitor [278]. For regenerative grafts, this provides proof that a poxviral multi-ligand architecture can be reduced to a synthetic biologic. Whether such combined control should be produced continuously by a living graft is a separate question.

Finally, if inflammatory and recognition barriers are insufficient, terminal cytoprotection can be placed at different death-execution levels. B13R, F1L, CrmA, and M1 act at non-equivalent points spanning inflammatory or extrinsic caspases, mitochondrial commitment, and apoptosome output [221,222,224,226]. The useful lesson is to identify the death pathway that actually limits the final graft and protect that level, rather than creating generic apoptosis resistance.

The regenerative-graft architecture is therefore also conditional. T/NK control follows antigen-presentation attenuation; interface, IFN, chemokine, inflammatory-signaling, and cytoprotective modules remain independent options chosen according to the final lineage, implantation route, and tissue environment.

### 7.4. Product-Level Evidence and Control Gates

A new module should first address a mechanism shown to limit the intended final product. Viral studies establish what a poxviral mechanism does; human-pathway perturbations show what happens when the corresponding host node is altered; final-product studies determine whether the intervention is compatible with the therapeutic cell.

Protection and function must be tested together. CAR-T and CAR-NK products must retain trafficking, CAR–target conjugation, repeated killing, expansion, required cytokine responses, and depletion. Regenerative grafts must retain differentiation, maturation, tissue-specific function, vascular integration, and metabolic stability.

Validation should reproduce the relevant route, tissue, and stress environment. Human immune diversity is required when HLA, KIR, antibodies, complement, or myeloid responses determine the outcome. The implementation itself must also be defined: molecular identity, localization, diffusion, duration, reversibility, expression heterogeneity, and stability are part of the intervention.

Finally, independent elimination must remain functional after all immune-evasion modifications. Together, these gates define a conditional defense-in-depth roadmap: a module is justified only when it closes a demonstrated residual barrier while the final product remains functional and independently controllable.

The available evidence does not define a universal minimum combination of immune-evasion interventions or a fixed number of necessary layers. Studies differ in therapeutic lineage, recipient immunity, administration site, conditioning, and duration of observation [5,7,8,57,63,70]. Consequently, combinations effective in one setting cannot be assumed sufficient in another, and Tier 1–3 assignments should not be interpreted as validated combination regimens. The endpoint is clinically useful persistence with preserved therapeutic function and reliable control. Additional complexity is justified only by demonstrable incremental benefit in the intended product; marginal gains in persistence do not by themselves outweigh impaired function, greater manufacturing uncertainty, or reduced eliminability. This provides a stopping criterion for the conceptual framework while acknowledging that sufficient combinations remain an empirical, product-specific question.

## 8. Safety and Evidence Boundaries

Immune protection should not be considered successful if it compromises the therapeutic function of the product. For CAR-based cells, protection must preserve trafficking, synapse formation, repeated killing, expansion, cytokine responsiveness, and rapid depletion. For iPSC-derived regenerative grafts, it must preserve differentiation, maturation, tissue-specific function, vascular integration, and long-term graft survival.

The second boundary is excessive immune escape. A mechanism that protects the intended therapeutic cell may also protect a damaged, infected, misplaced, growth-advantaged, or transformed cell. As endogenous routes of surveillance are removed, independent detection and elimination become more important.

Independent detection requires clinical surveillance tailored to the product and implantation site. For circulating CAR-T products, cell enumeration and transgene measurements can document persistence and expansion, although these measurements alone cannot distinguish therapeutic activity from abnormal cellular behavior [13]. Implanted regenerative grafts require assessment of tissue-specific function together with appropriate imaging. For example, the clinical study of allogeneic iPSC-derived dopaminergic grafts used MRI to assess graft overgrowth and PET to evaluate dopaminergic activity [12]. Unexplained expansion, altered graft morphology, or deteriorating function would warrant diagnostic investigation, including tissue assessment when clinically feasible. Neither blood monitoring nor imaging guarantees detection of small, ectopic, or otherwise occult abnormal populations. Follow-up for persistent genetically modified products should also address delayed adverse events through a risk-based surveillance plan [289].

Triggered elimination is a separate safeguard. Clinical depletion of modified donor T cells using an inducible caspase-9 safety switch establishes a precedent for active intervention after cell administration [100]. Evidence in CAR-T models and iPSC-derived systems is preclinical or in vitro [284,286], and variable sensitivity in human iPSCs demonstrates that switch expression alone does not guarantee complete clearance [290]. Thus, the presence of an elimination construct cannot substitute for clinical monitoring or establish lifelong control; reliable detection and effective elimination remain separate requirements.

### 8.1. Orthogonal Elimination

A product cannot rely for safety on the same mechanism that it has been engineered to evade. This applies to both the trigger of elimination and the execution pathway.

For example, an antibody-dependent depletion marker may be independent of HLA recognition but could still be weakened by engineering that blocks Fc- or complement-dependent clearance. Likewise, an inducible apoptosis switch may have an independent trigger but still depend on caspase or mitochondrial machinery altered by survival engineering.

This distinction should be tested experimentally in the final product. Engineered SERPINB9 provides a useful example: selected endogenous killing pathways were attenuated while inducible depletion remained functional in the tested CAR-T system [74]. By contrast, deeper interventions at mitochondrial or apoptosome levels may overlap more directly with apoptosis-based safety systems.

An elimination system should therefore be evaluated after all stealth modifications, after activation or differentiation, under inflammatory stress, and across relevant product subpopulations. iCasp9 provides an important precedent, but incomplete expression, silencing, altered execution, or switch-negative cells remain possible failure modes [284,286].

### 8.2. Localization, Duration, and Immunogenicity

The range of an intervention matters as much as its molecular target. A surface-retained IFN or complement regulator, a graft pretreatment, a transient recombinant protein, and a constitutively secreted transgene can act on the same pathway yet produce markedly different biological consequences.

This is especially important for extracellular poxviral mechanisms. B18/B19R, M-T7, VCP, cytokine decoys, and chemokine-binding proteins can alter the local environment rather than only the engineered cell [124,220,246]. Their concentration, diffusion, duration, and effects on neighboring cells should therefore be treated as part of the mechanism.

An introduced viral protein may itself become a target for recipient immunity. If relevant peptides are presented by the therapeutic cell, protein-specific CD8^+^ T cells may contribute to its elimination; antigen acquisition by recipient APCs can also sustain responses despite reduced donor-cell presentation [280,281]. Clinical immune responses to CAR-derived sequences illustrate the general risk, without predicting the immunogenicity of a particular poxviral protein [13]. Protein deimmunization is therefore an investigational option with a substantial development burden: reduced predicted antigenicity does not establish clinical tolerance or preserved activity. This uncertainty increases when several foreign proteins are introduced [280,281].

### 8.3. The Final Product Is the Relevant Validation Unit

Activity in an infected cell, reporter assay, parental iPSC clone, or resting immune cell does not establish compatibility with a final therapeutic product. Differentiation, expansion, cryopreservation, repeated CAR activation, inflammatory exposure, and implantation can change HLA expression, NK ligands, adhesion molecules, cytokine output, transgene abundance, and death sensitivity.

Product heterogeneity matters for the same reason. Small TCR-positive, HLA-replete, transgene-negative, safety-switch-negative, incompletely differentiated, or growth-advantaged populations may behave differently from the bulk product. Product characterization should therefore connect immune-protection activity with identity, purity, editing completeness, genome integrity, expression stability, therapeutic function, and elimination competence [8,291].

Animal models should be interpreted only for the biology they reproduce. Species-specific cytokine, complement, or receptor interactions can change the apparent activity of a poxviral mechanism. Human final-lineage cells and donor-diverse human immune systems therefore remain necessary before assigning final-product relevance.

The general rule is simple: the broader, longer-lived, and less localized the protection, the more independent its control must be.

## 9. Conclusions and Outlook

Current hypoimmune engineering is most advanced for direct HLA-dependent recognition. Important residual barriers remain in recipient-APC presentation, NK-cell diversity, humoral responses, blood- and tissue-interface injury, inflammatory remodeling, and terminal killing. Poxviruses are useful because they separate these pressures into distinct control points rather than treating immune escape as a single phenotype.

Several design lessons emerge. IFN can be controlled at different extracellular and intracellular levels. Antigen presentation can be tuned through peptide processing and protein trafficking rather than only through HLA deletion, but any reduction in HLA-I or peptide supply must be considered together with NK-cell recognition. Extracellular protection can also be spatial: poxviruses control cytokine fields, chemokine positioning, and complement close to the protected cell. Finally, viral survival proteins show that terminal killing can be intercepted at several non-equivalent execution points.

Some of these ideas have already moved beyond virology. The 018–STAT1 interface has inspired synthetic peptide mimetics, the CrmB/CrmD architecture has inspired a bifunctional TNF/chemokine biologic, and host-derived surface complement regulators reproduce the spatial logic of poxviral complement protection [277,278,287]. Other concepts, particularly selective post-assembly HLA trafficking and local control of chemokine–GAG gradients, remain underexplored in low-immunogenic cell products.

Direct expression of an intact poxviral protein is therefore only one translational route. Future studies should separate molecular identity from exposure format: native viral factor versus a minimized, deimmunized, or host-derived functional analogue, and constitutive versus transient, localized, or inducible activity. These comparisons should be performed in the same final therapeutic lineage and should assess immune protection together with product function, bystander effects, immunogenicity, expression stability, and independent elimination.

The desired endpoint is controlled low immunogenicity, not universal immune invisibility.

## Figures and Tables

**Table 2:** Poxviral IFN-evasion principles, human intervention evidence, and product-specific interpretation.

Intervention Level	Poxviral Principle	Human Intervention Evidence	CAR-Product Interpretation	iPSC-Derived Graft Interpretation	Tier/Main Constraint
Upstream IFN induction	Poxin and E5 suppress cGAS–STING; C4/C16 interfere with DNA-PK/Ku; C6/K7/N2 inhibit the TBK1–IRF axis [110,115,116,117].	Human DNA-PK has an innate sensing function in addition to DNA repair. TBK1 deficiency can increase TNF-driven cell death [121,122]. No direct final-product evidence supports broad stable suppression.	Mainly a stress- and safety-validation axis rather than a preferred stealth edit.	Stable DNA-PK/Ku suppression is poorly suited to clonally expanded grafts because of its genome-maintenance role.	Tier 3. Main concerns are genome integrity, antiviral defense, and stress signaling.
Extracellular IFN buffering	B18/B19R traps type I IFNs and can remain surface-associated; B8-like receptors trap IFN-γ [111,112,124].	B18R protein or mRNA suppresses IFN-I responses in human-cell RNA-delivery and reprogramming systems [125,127]. In human CAR-T cells, IFN-γ availability has also been altered directly by neutralizing antibody or IFNG knockout; both approaches preserved antitumor activity in hematologic models while reducing macrophage activation [142].	The effect of extracellular cytokine control depends on signal strength, duration, and disease compartment. Temporary IFN-I exposure can improve CAR-T manufacturing, whereas persistent IFN-I can promote dysfunction. IFN-γ neutralization may be compatible with hematologic CAR-T activity, but a diffusible IFN-γ trap can also alter tumor and stromal compartments.	Strongest fit is local or transient buffering during inflammatory stress or early engraftment.	Tier 2 when local or reversible. Bystander and antiviral effects must be controlled. Poorly confined IFN-γ trapping can approach Tier 3.
IFN receptor responsiveness	B18/B8 prevent receptor engagement; 018 blocks receptor-proximal STAT1 recruitment [130].	Antibody-mediated IFNAR blockade reduces chronic IFN-I-associated dysfunction and improves cytotoxicity in human CAR-T cells [140]. IFNGR1 disruption reduces IFN-γ-induced death and improves expansion or persistence of CD28 CAR-T cells [143]. Target-cell IFNγR signaling can nevertheless support CAR–target adhesion and killing in solid tumors [129].	Product-restricted receptor tuning can protect the CAR cell without necessarily changing IFN signaling throughout the tissue. The target-cell compartment must be evaluated separately.	Receptor attenuation may reduce inflammatory reprogramming, but the effect must be tested in the final differentiated lineage.	Usually Tier 3 if permanent. Main concerns are antiviral defense and loss of beneficial IFN functions.
JAK-level signal attenuation	VH1 and C6 show that IFN signaling can be interrupted downstream of receptor engagement [113,114].	TYK2 silencing, knockout, or inhibition reduces IFN-α responses in human β cells and stem-cell-derived endocrine models; complete loss can affect development [87,137,138].	Broad JAK blockade is difficult to reconcile with cytokine-dependent CAR function.	Reversible TYK2 attenuation has direct endocrine relevance and is more attractive than broad JAK/STAT loss.	Reversible TYK2 tuning: Tier 2. Broad JAK/STAT blockade: Tier 3.
Endogenous negative feedback	Poxviral IFN antagonism shows the value of limiting signal strength and duration; host feedback provides a non-viral route to the same regulatory problem.	SOCS1/3 gain- and loss-of-function studies demonstrate bidirectional control of human IFN/JAK/STAT output, and high endogenous SOCS1 contributes to attenuated IFN-β responsiveness in human pluripotent cells [144,145]. PTPN2 loss worsens inflammatory signaling and maturation in human stem-cell-derived islets but enhances several functional properties of human CAR-T cells; adenoviral PTPN2 overexpression provides reciprocal human β-cell evidence for protection from ER-stress-induced death [146,147,148].	Reinforcing broad JAK/STAT negative feedback may oppose cytokine-dependent CAR activation and expansion; PTPN2 loss instead illustrates how releasing this brake can improve selected CAR-T functions.	Preserving or selectively reinforcing endogenous feedback may be more compatible with long-lived graft protection than removing these brakes, but direct stable gain-of-function evidence in final differentiated iPSC-derived grafts is not yet available.	Tier 2 as a feedback-tuning concept; Tier 3 for broad constitutive reinforcement without final-product validation. Main constraints are cytokine-pathway breadth, lineage function, antiviral competence, and reversibility.

Tier 1–3 is a qualitative translational scale used throughout the review. Tier 1 denotes approaches close to established cell-therapy engineering; Tier 2 denotes mechanisms with strong supporting evidence that still require product-specific validation; and Tier 3 denotes broad or pleiotropic interventions with a high control burden. Downstream PKR, OAS/RNase L, ZBP1–RIPK3–MLKL, and SAMD9/SAMD9L pathways are treated mainly as antiviral-competence and safety readouts rather than preferred IFN-engineering targets.

**Table 3:** Poxviral adaptive immune-control principles, human intervention evidence, and product-specific interpretation.

Control Point	Poxviral Principle	Human/Product Evidence	Main Observed Effect	CAR Interpretation	iPSC-Graft Interpretation	Tier/Main Constraint
**MHC-I peptide transport**	**CPXV012** blocks TAP-dependent peptide transport [149,152].	TAP1/TAPBP disruption in hESCs; TAP2-deficient human iPSCs and iPSC-derived myeloid cells; BNLF2a CAR-T comparator [63,162,163].	↓ surface HLA-I and/or ↓ alloreactive CD8^+^ recognition in the tested systems. TAP-dependent HLA-E peptide supply can also fall [154].	Direct CAR comparator supports peptide-supply tuning, but NK protection must be retained.	Demonstrates iPSC-derived-cell tractability, not yet a validated regenerative-graft strategy; recipient-APC presentation persists.	**Tier 2.** Main constraint: coupled classical HLA-I/HLA-E effects and residual indirect presentation.
**MHC-I processing program, peptide loading and repertoire editing**	**MC080** degrades tapasin and destabilizes the peptide-loading complex [150].	TAPBP hESC disruption; NLRC5 program-level regulation; TAPBPR and ER-processing perturbations; ERAP1/2 loss; PDIA3-edited human CAR-T cells [162,164,165,166,167,168].	Perturbations produce non-equivalent changes in HLA abundance and immunopeptidome; ERAP1/2 loss can weaken HLA-E/NKG2A inhibition, while PDIA3 loss increased CAR effector activity.	ER-processing genes are not interchangeable stealth targets; product function may change independently of HLA.	Peptide-repertoire tuning may alter both CD8 recognition and NK inhibition; differentiated-lineage evidence remains limited.	**Tier 2, high caution.** Main constraint: unpredictable immunopeptidome, HLA-E and product-function effects.
**MHC-I surface trafficking and composition**	**CPXV203** retrieves mature MHC-I away from surface display [151,172].	K3/K5 and controlled Nef in human T/CAR cells; selective HLA and HLA-E engineering in human PSC systems [36,57,70].	Graded or selective reduction in surface HLA can decrease T-cell recognition without requiring complete pan-HLA loss.	Strongest direct evidence favors partial/selective HLA control with retained NK-inhibitory input.	Selective HLA composition is preferable to complete HLA-I erasure when durable NK tolerance is required.	**Host strategies: Tier 1; CPXV203 principle: Tier 2.** Main constraint: recipient priming and NK missing-self.
**MHC-II presentation and CD4^+^ recognition**	**A35R** attenuates MHC-II-restricted presentation; direct host target unresolved [158].	CIITA suppression in CAR-T cells and B2M/CIITA-edited hPSC platforms; inflammatory human β-cell data identify HLA-II, CD74 and peptide-loading machinery as downstream nodes [38,39,45,63].	↓ donor-cell HLA-II-dependent recognition after CIITA control; inflammatory exposure can induce additional MHC-II machinery.	Useful for CD4^+^ and anti-transgene T-cell pressure, but does not prevent recipient-APC presentation.	Relevant to inflammation-inducible HLA-II in differentiated grafts; indirect CD4^+^ recognition remains.	**A35R: Tier 2; CIITA strategies: Tier 1/2.** Main constraint: recipient-compartment presentation.
**Costimulatory and inhibitory T-cell interfaces**	**M2** sequesters CD80/CD86 and interferes with CD28/CTLA-4 interactions; recombinant oligomeric M2 suppresses primary human T-cell activation outside infection [159,173].	Belatacept/CTLA4-Ig validates B7 blockade; CTLA4-Ig + PD-L1 protected differentiated hESC-derived grafts, including lineage-restricted hepatocyte-like products [169,170,171].	B7 blockade lowers positive costimulation; combined inhibitory-checkpoint and costimulatory control can produce local graft protection.	M2 would not directly block an intracellular CD28 CAR domain, but broad extracellular B7 manipulation can alter endogenous T-cell/APC regulation.	Localized modulation of APC–T-cell or inhibitory-checkpoint interfaces has direct graft-proximal precedent.	**M2: Tier 2; localized host circuits: Tier 1/2.** Main constraint: localization and non-equivalence of CD28, CTLA-4 and PD-1 pathways.
**IgG–FcγR-linked adaptive amplification**	**CPXV14** binds FcγRs and suppresses antibody-supported T-cell activation through FcγR-positive accessory cells [160].	Human FcγR biology supports immune-complex uptake/presentation; therapeutic-cell CD64 captures IgG and protects CAR-T and iPSC-derived cells from antibody-dependent injury [44,174].	CPXV14 reduces FcγR-supported activation; CD64 reduces downstream IgG-mediated ADCC/CDC by a distinct mechanism.	Relevant to anti-HLA, anti-CAR and other product antibodies; CD64 has direct product evidence.	Relevant to antibody-bound graft material; CD64 protects from downstream injury but does not prevent antibody formation.	**CPXV14: Tier 2; CD64: Tier 1.** Main constraint: FcγRs are compartment-specific and CD64 is a functional comparator, not a molecular mimic.
**Responder T-cell responsiveness**	**B22 family** suppresses TCR-dependent human T-cell activation after receptor engagement; precise host target remains unresolved [161].	Viral ectopic-expression and deletion studies establish the phenotype; no corresponding host-gene intervention is proposed.	Broad suppression of responder T-cell activation.	Poorly aligned with an immune-effector product that requires intact activation and cytotoxicity.	Would suppress host T cells rather than selectively reduce graft visibility and does not control other rejection pathways.	**Tier 3 boundary mechanism.** Main constraint: unresolved target, broad responder-cell suppression and poor product selectivity.

Tier 1–3 is a qualitative translational scale used throughout the review. Tier 1 denotes interventions already close to established cell-therapy engineering or supported directly in relevant therapeutic products; Tier 2 denotes strong mechanistic and human-cell evidence requiring product-specific validation; Tier 3 denotes broad, pleiotropic, unresolved, or poorly product-compatible mechanisms with a high control burden. Host intervention nodes listed alongside a poxviral principle are not assumed to be direct molecular targets of that viral protein. Symbols: ↓, decrease; arrows indicate the direction of the reported effect rather than a uniform change across all models.

**Table 4:** Poxviral NK-control principles, human intervention evidence, and product-specific interpretation.

Control Point	Poxviral Principle	Human/Product Evidence	Main Observed Effect	CAR Interpretation	iPSC-Graft Interpretation	Tier/Main Constraint
**Inhibitory NK signaling after HLA reduction**	Poxviral activating-receptor evasion acts on top of, rather than replaces, the inhibitory HLA background.	HLA-E expression protects B2M-edited hPSCs; selective HLA-A/B disruption preserves HLA-C; HLA-E SCT and LLT1 provide CAR-level inhibitory strategies [36,37,69,187].	Selected inhibitory signals reduce NK attack without restoring the complete HLA repertoire.	Closest to current CAR engineering; inhibitory input must not interfere with CAR function.	Useful after HLA reduction, but stability must be confirmed in the differentiated graft.	**Tier 1–2.** Main constraint: recipient receptor repertoire and stability of inhibitory-ligand expression.
**NKG2D-ligand composition and surface display**	OMCP blocks the NKG2D receptor interface and separates induced-self recognition from missing-self recognition [58,175].	MICA/MICB were removed within a multiplex-edited hPSC platform containing additional HLA-I/HLA-II edits [191].	Direct ligand removal is feasible, but its isolated contribution to graft protection was not resolved.	Broad NKG2D blockade is poorly aligned with antitumor and antiviral surveillance; ligand-selective approaches require direct CAR evidence.	Demonstrates hPSC tractability, not protection of a differentiated human allograft.	**Tier 2.** Main constraint: stress and abnormal-cell surveillance.
**NKG2D-ligand transcriptional control**	OMCP identifies NKG2D-ligand availability as a controllable upstream variable.	CREBBP/CBP and EP300/p300 double loss in human HEK-293 cells reduced basal and stress-induced MICA/B and ULBP2 [188].	NKG2D-ligand expression and primary-NK-cell killing decreased.	Mechanistic evidence only; CBP/p300 are too pleiotropic to treat as simple CAR stealth targets.	Useful as a stress-ligand regulatory axis, not as a validated graft edit.	**Tier 2 mechanistic evidence; Tier 3 for broad stable CBP/p300 suppression.**
**NKG2D-ligand shedding and extracellular availability**	OMCP blocks receptor engagement; host studies show that ligand localization can change the same interface in the opposite direction.	Decitabine increased TIMP3 and reduced ADAM17-dependent MICA/MICB/ULBP2 shedding in human AML cells; anti-ULBP6/2/5 provides an extracellular-ligand intervention [189,190].	Less shedding increased surface NKG2D recognition and NK cytotoxicity; soluble-ligand neutralization has additional Fc-dependent effects.	Demonstrates why manipulating soluble ligands is not equivalent to reducing target-cell recognition.	Surface and soluble ligand states should be distinguished under graft stress.	**Tier 2 validation axis.** Main constraint: direction of effect can reverse with ligand localization.
**NKp30-ligand composition and extracellular form**	HA inhibits NKp30-triggered activation [59].	NCR3LG1/B7-H6 deletion reduced NKp30-dependent killing of activated T/CAR-T cells; EV-associated and soluble BAG6 have different NKp30 effects [183,184,185,192].	B7-H6 loss reduced NK surveillance and improved CAR-T proliferation/persistence; BAG6 activity depended on extracellular form.	B7-H6 ligand editing is substantially closer to product engineering than receptor-wide NKp30 inhibition.	Differentiation can introduce B7-H6/NKp30 pressure; BAG6 must be assessed by form and localization.	**HA principle: Tier 2; B7-H6 comparator: Tier 1–2.** Main constraint: preserve broader NK surveillance.
**NKp46 stress-ligand surface exposure**	HA stimulates NKp46-dependent signaling, showing that NKp46 cannot be inferred from NKp30 behavior [59].	Ecto-CRT on senescent human A549 cells was functionally blocked with antibody; NCR1/NKp46 loss provided concordant evidence [186].	Blocking the ecto-CRT–NKp46 interaction reduced NK-cell killing.	Primarily a stress-surveillance readout; no direct CAR-stealth validation.	ER stress can expose ecto-CRT after differentiation or implantation, but suppressing it may protect abnormal cells.	**Tier 2 interface; receptor-wide suppression: Tier 3.** Main constraint: abnormal-cell surveillance.
**Adhesion/contact and antibody/CD16 execution**	CPXV14 illustrates FcγR-linked immune amplification; adhesion control is represented by host comparators [160].	CD54/CD58 deletion reduced host-cell attack against engineered CAR products; CD64 reduced IgG-dependent injury [44,73].	Productive cytotoxic contact or antibody-dependent effector injury decreased.	Potentially useful, but CAR–target adhesion, immune-synapse formation, and repeated killing must remain intact.	Relevant where inflammatory adhesion or alloantibody exposure contributes to graft injury; direct regenerative-product evidence is less complete.	**Host comparators: Tier 1–2; Fc poxviral bridge: Tier 2.** Main constraint: therapeutic contact and antibody biology.

Tier 1–3 is the qualitative translational scale used throughout the review. Tier 1 denotes approaches close to established cell-therapy engineering; Tier 2 denotes mechanisms with strong mechanistic or human-cell support that still require product-specific validation; and Tier 3 denotes broad or pleiotropic interventions with a high control burden.

**Table 5:** Poxviral inflammatory-survival control points, human intervention evidence, and product-specific interpretation.

Control Point	Poxviral Principle	Human/Product Evidence	Main Observed Effect	CAR Interpretation	iPSC-Graft Interpretation	Tier/Main Constraint
**Extracellular TNF field/TNFR response**	CrmE captures TNF; CrmB/CrmD add SECRET-domain chemokine binding [196,200,201,272].	Etanercept improved rat hepatocyte engraftment; sTNFRII–Fc protected human MSCs; TNFR1 attenuation lowered TNF-induced HLA-I/adhesion in HUVECs, whereas TNFR2 loss impaired MSC regulatory/angiogenic functions [229,230,231,233].	Extracellular capture reduces inflammatory recruitment; responder tuning can alter both visibility and cell function.	Prefer selective responder/source control to a chronic diffusible sink.	Local/peri-engraftment buffering is plausible; preserve TNFR2-linked repair.	Local/reversible interception **Tier 2**; stable receptor loss or broad Crm-like secretion **Tier 3**.
**Extracellular IL-1 control**	B15/B16-like proteins capture IL-1β before receptor engagement [198,273].	IL1RN protected human islets; IL1R2 acted as a decoy; IL1RAP blockade reduced IL-1-family signaling, adhesion molecules, permeability, and neutrophil adhesion in human endothelium [235,236,237,274].	Extracellular or receptor-complex control lowers IL-1-driven injury without deleting the entire pathway.	Recipient/local extracellular control is usually more defensible than routine CAR-cell receptor deletion.	Best fit is local or short peri-engraftment buffering.	Local/reversible **Tier 2**; broad permanent responder blockade **Tier 2/3**.
**Extracellular IL-18 control**	C12 acts as an IL-18-binding protein and reduces NK/T-cell–IFN-γ amplification [199,275].	Tadekinig alfa demonstrates reversible IL18BP pharmacology; IL-18 armoring can enhance CAR activity, whereas IL18BP can restrict it [238,239,240].	The same extracellular intervention can be anti-inflammatory or anti-therapeutic.	Constitutive IL-18 trapping is usually a poor fit.	Local buffering is plausible but lacks mature final-product evidence.	Graft-local concept **Tier 2**; broad CAR trapping **Tier 3/negative fit**.
**GM-CSF source versus responder control**	ORFV GIF is a species-restricted precedent for extracellular GM-CSF capture [202,203].	CSF2 loss/neutralization reduced CAR-driven monocyte cytokines; CSF2RA-deficient iPSC macrophages lost STAT5 signaling and phagocytic function [241,242,243,244].	Source control reduces paracrine myeloid amplification; responder loss can remove intended lineage function.	Direct evidence supports CAR-cell CSF2 source control.	Preserve GM-CSF signaling in macrophage-lineage products.	CAR source control **Tier 1–2**; broad neutralization **Tier 2**; receptor deletion **Tier 3**.
**Chemokine availability and GAG-dependent positioning**	vCCI/35K and M-T1 block receptor engagement; M-T7, A41, and E163 disrupt chemokine–GAG presentation [204,205,206,207,209].	M-T7 reduced graft infiltration/pathology with model-dependent survival effects; membrane 35K showed discordant in-vivo activity; CXCL10 loss partly protected human SC-islets [53,88,245,246,248].	Recruitment falls only when the intervention reaches the active tissue gradient.	Broad sequestration risks impaired tumor homing and tissue entry.	Local or ligand-selective control is plausible; preserve vascularization.	Spatially validated local/selective control **Tier 2**; long-lived broad sinks **Tier 3**.
**Cell-proximal complement/blood interface**	VCP/SPICE restrict C3/C4 amplification; A56 retains VCP near the surface [218,219,220].	Recombinant VCP protected graft-relevant interfaces; CD55 reduced complement deposition in human iPSC kidney organoids; CFH overexpression reduced MAC deposition in iPSC-derived choroidal endothelium; CD64 and APT070 reduced IgG/complement injury [44,83,84,251,252,254].	Local surface/pericellular regulation reduces selected complement/Fc injury but not antibody formation.	Useful when humoral clearance is demonstrated; not a universal CAR module.	Strong fit for blood-contacting, endothelial, vascularized, or islet-like products.	Host-derived local modules **Tier 1–2**; poxviral complement principle **Tier 2**; broad systemic suppression **Tier 3**.
**TLR/IL-1R–NF-κB signaling threshold**	A46/A52 act proximally; MC005/MC008 converge on NEMO; B14 inhibits IKKβ; A49 targets IκB turnover; F14 alters p65–CBP/p300 output. A52 also disrupts autophagic flux [211,212,213,214,215,216,217].	A20 overexpression protected experimental islets, whereas NF-κB signaling supports CAR activation and persistence [89,258].	Feedback tuning can lower inflammatory output while preserving core pathway function.	Central blockade is poorly aligned with CAR activation.	Threshold tuning is more plausible than pathway deletion.	Feedback-based tuning **Tier 2**; permanent central blockade **Tier 3**.
**Inflammasome/pyroptotic amplification**	B13R links poxviral evasion to caspase-1/IL-1β processing [222].	CAR-associated pyroptosis can arise from target-cell GSDME and recipient macrophages; GSDMD- and AIM2-associated programs also localize to recipient inflammatory macrophages in cardiac rejection models [98,259,260,261].	The dominant causal compartment may lie outside the therapeutic cell.	Routine CAR-cell inflammasome editing is unsupported when target/recipient cells dominate.	Local or recipient-directed control may help after compartment mapping.	Compartment-defined transient control **Tier 2**; cell-type-agnostic permanent ablation **Tier 3**.
**Extrinsic and granule-mediated death execution**	B13R/CrmA inhibit inflammatory or extrinsic caspase pathways; CrmA has autonomous graft-protective activity outside infection [221,222,223,263,264].	FAS disruption/dominant-negative FAS improved CAR resistance; engineered SERPINB9 reduced granzyme/Fas-associated elimination while preserving inducible depletion [48,74,262].	Selective execution resistance can improve persistence without suppressing upstream recognition.	Strong direct product evidence; preserve repeated killing and depletion.	Broad caspase resistance could impair removal of damaged or abnormal cells.	Selective CAR protection **Tier 1–2**; broad CrmA/FADD/CASP8-like resistance **Tier 3**.
**Mitochondrial commitment/apoptosome output**	F1L limits mitochondrial commitment; P1L binds BID; M1 limits apoptosome output; N1 remains disputed [224,225,226,227,228].	BAX/BAK1 DKO human iPSCs gained stress resistance but developed mitochondrial/developmental abnormalities after neural differentiation; human-islet and CAR studies show context-dependent effects of XIAP/BCL2-family protection [265,266,267,268,276].	Deep resistance may improve persistence but can prolong toxicity or impair depletion.	Deep mitochondrial or apoptosome resistance is poorly aligned with routine CAR engineering because it may prolong toxicity and interfere with apoptosis-based depletion.	High risk for residual progenitors, dysfunctional cells, or abnormal clones.	Permanent mitochondrial/apoptosome resistance Tier 3; independent eliminability required.

Tier 1–3 is the qualitative translational scale used throughout the review. Tier 1 denotes approaches close to established cell-therapy engineering or supported directly in the intended product; Tier 2 denotes mechanisms with strong mechanistic, human-cell, or product-proximal support that still require final-product validation; Tier 3 denotes broad, pleiotropic, or difficult-to-reverse interventions with a high functional or control burden. Poxviral infection or animal-transplantation evidence establishes mechanism or transplantation relevance but is not treated as final human-product evidence.

## Data Availability

No new data were created or analyzed in this study. Data sharing is not applicable to this article.

## Abbreviations

The following abbreviations are used in this manuscript:

A20	tumor necrosis factor alpha-induced protein 3 (TNFAIP3)
ADCC	antibody-dependent cellular cytotoxicity
AML	acute myeloid leukemia
AP-1	activator protein 1
APC	antigen-presenting cell
APT070	mirococept, a membrane-localizing C3-convertase inhibitor
ATP	adenosine triphosphate
B2M	beta-2-microglobulin
B7-H6	B7 homolog 6 (NCR3LG1)
CAR	chimeric antigen receptor
CAR-NK	chimeric antigen receptor-engineered natural killer cell
CAR-T	chimeric antigen receptor T cell
Cas9	CRISPR-associated protein 9
CBP	CREB-binding protein
CD	cluster of differentiation
CDC	complement-dependent cytotoxicity
CFH	complement factor H
cGAMP	2′3′-cyclic GMP–AMP
cGAS	cyclic GMP–AMP synthase
CIITA	class II major histocompatibility complex transactivator
CPXV	cowpox virus
Crm	cytokine response modifier
CTLA-4	cytotoxic T-lymphocyte-associated protein 4
CTLA4-Ig	cytotoxic T-lymphocyte-associated protein 4–immunoglobulin fusion protein
DAMP	damage-associated molecular pattern
DKO	double knockout
DNA	deoxyribonucleic acid
DNA-PK	DNA-dependent protein kinase
EBV	Epstein–Barr virus
ecto-CRT	ecto-calreticulin
ER	endoplasmic reticulum
EV	extracellular vesicle
Fc	fragment crystallizable region
FcγR	Fc gamma receptor
GAG	glycosaminoglycan
GAF	gamma-activated factor
GIF	GM-CSF- and IL-2-inhibitory factor
GM-CSF	granulocyte–macrophage colony-stimulating factor
GPVI	glycoprotein VI
GVHD	graft-versus-host disease
HA	hemagglutinin
HHV-8	human herpesvirus 8
HIP	hypoimmune
HIV-1	human immunodeficiency virus type 1
HLA	human leukocyte antigen
HLA-I/HLA-II	human leukocyte antigen class I/class II
hESC	human embryonic stem cell
hPSC	human pluripotent stem cell
HUVEC	human umbilical vein endothelial cell
IBMIR	instant blood-mediated inflammatory reaction
ICAM-1	intercellular adhesion molecule 1 (CD54)
IFN	interferon
IFN-I	type I interferon
IFN-α/β	interferon alpha/beta
IFN-γ	interferon gamma
IFNAR/IFNAR1/2	interferon alpha/beta receptor/receptor subunits 1 and 2
IFNGR/IFNGR1/2	interferon gamma receptor/receptor subunits 1 and 2
IgG	immunoglobulin G
IgM	immunoglobulin M
IKK	IκB kinase
IKKε	IκB kinase epsilon
IL	interleukin
IL-1R	interleukin-1 receptor
iCasp9	inducible caspase-9
iPSC	induced pluripotent stem cell
IRF	interferon regulatory factor
ISG	interferon-stimulated gene
ISGF3	interferon-stimulated gene factor 3
IκB	inhibitor of κB
JAK/STAT	Janus kinase/signal transducer and activator of transcription
KIR	killer-cell immunoglobulin-like receptor
LFA-1	lymphocyte function-associated antigen 1
LLT1	lectin-like transcript 1 (CLEC2D)
MAC	membrane attack complex
MAPK	mitogen-activated protein kinase
MCV	molluscum contagiosum virus
MHC	major histocompatibility complex
MHC-I/MHC-II	major histocompatibility complex class I/class II
MICA/MICB	MHC class I polypeptide-related sequence A/B
MPXV	monkeypox virus
mRNA	messenger RNA
MSC	mesenchymal stromal cell
NCR	natural cytotoxicity receptor
NEMO	NF-κB essential modulator (IKKγ)
NET	neutrophil extracellular trap
NF-κB	nuclear factor kappa B
NK	natural killer
NKG2A/NKG2C/NKG2D	natural killer group 2A/2C/2D receptors
NKp30	natural cytotoxicity triggering receptor 3 (NCR3)
NKp46	natural cytotoxicity triggering receptor 1 (NCR1)
NLRC5	NLR family CARD domain-containing 5
OMCP	orthopoxvirus MHC class I-like protein
ORFV	orf virus
p300	E1A-binding protein p300 (EP300)
PD-1	programmed cell death protein 1
PD-L1	programmed death-ligand 1
PEBL	protein-expression blocker
PMN	polymorphonuclear neutrophil
PSC	pluripotent stem cell
PTPN2	protein tyrosine phosphatase non-receptor type 2
RNA	ribonucleic acid
RPE	retinal pigment epithelium
SC-islet	stem-cell-derived islet
SCT	single-chain trimer
SECRET	smallpox virus-encoded chemokine receptor domain
SOCS1/SOCS3	suppressor of cytokine signaling 1/3
SPICE	smallpox inhibitor of complement enzymes
STING	stimulator of interferon genes
TAP	transporter associated with antigen processing
TAPBP/TAPBPR	TAP-binding protein (tapasin)/TAP-binding protein-related
TCR	T-cell receptor
TLR	Toll-like receptor
TNF	tumor necrosis factor
TNFR	tumor necrosis factor receptor
TYK2	tyrosine kinase 2
ULBP	UL16-binding protein
VACV	vaccinia virus
VCP	vaccinia complement control protein
vCCI	viral CC-chemokine inhibitor
VICE	vaccinia inhibitor of complement enzymes
